# Hierarchically self-assembled homochiral helical microtoroids

**DOI:** 10.1038/s41565-022-01234-w

**Published:** 2022-11-03

**Authors:** Cong Du, Zujian Li, Xuefeng Zhu, Guanghui Ouyang, Minghua Liu

**Affiliations:** 1 Beijing National Laboratory of Molecular Sciences and CAS Key Laboratory of Colloid, Interface and Thermodynamics, Institute of Chemistry, Chinese Academy of Sciences, Beijing, China; 2 University of Chinese Academy of Sciences, Beijing, China

**Keywords:** Molecular self-assembly, Supramolecular polymers

## Abstract

Fabricating microscale helical structures from small molecules remains challenging due to the disfavoured torsion energy of twisted architectures and elusory chirality control at different hierarchical levels of assemblies. Here we report a combined solution–interface-directed assembly strategy for the formation of hierarchically self-assembled helical microtoroids with micrometre-scale lengths. A drop-evaporation assembly protocol on a solid substrate from pre-assembled intermediate colloids of enantiomeric binaphthalene bisurea compounds leads to microtoroids with preferred helicity, which depends on the molecular chirality of the starting enantiomers. Collective variable-temperature spectroscopic analyses, electron microscopy characterizations and theoretical simulations reveal a mechanism that simultaneously induces aggregation and cyclization to impart a favourable handedness to the final microtoroidal structures. We then use monodispersed luminescent helical toroids as chiral light-harvesting antenna and show excellent Förster resonance energy transfer ability to a co-hosted chiral acceptor dye, leading to unique circularly polarized luminescence. Our results shed light on the potential of the combined solution–interface-directed self-assembly approach in directing hierarchical chirality control and may advance the prospect of chiral superstructures at a higher length scale.

## Abstract (web summary)

Homochiral helical toroids with micrometre-scale lengths are successfully fabricated by a combined solution–interface-directed hierarchical self-assembly strategy.

## Main

Chirality is a universal phenomenon in nature and significantly affects the properties of both biomaterials and artificial materials^1–4^. The topology of hierarchical chiral architectures^5–8^, together with their chirality control across length scales^9^, plays a vital role in defining their diverse functionalities. Compared with widely reported chiral superstructures such as fibre bundles, tubes and helices^10–13^, self-assembled chiral toroidal structures remain largely unexplored due to their conformational constraints regarding circularity and helicity^14^. However, in biological systems, a hierarchical self-assembly mechanism involving the precise arrangement of building blocks and masterly control of chirality plays an important role in the formation of chiral circular bioarchitectures from nanoscales to macroscales. For example, the crystal structure of the light-harvesting antenna complex (LH2) shows that the protein subunits enclosing the pigment molecules unidirectionally assemble into helical ring-shaped topology, which contributes to exciton delocalization within the toroidal π-aggregates and enhances the energy transfer efficiency^15^. Another exquisite natural ring structure with larger size is the hierarchically organized coccolith skeletons wherein nanometre-sized calcium carbonate skeletal plates are helically oriented into micrometre toroids^16^. Inspired by these amazing naturally occurring helical toroids in living systems, the preparation of helical ring architectures has gained increasing interest among chemistry, materials and biology communities^17–23^. Despite the fact that the accurate asymmetric synthesis of homochiral molecular macrocycles or toroids has earned credit^24^, the development of their chiral supramolecular analogues at a higher level of hierarchy (such as micrometre scale) through a self-assembly approach is still in its infancy^25^, mainly due to the lack of efficient cyclization strategies and remaining challenges in the precise chirality control at higher length scales^9,26^.

Interfacial self-assembly has proven its capabilities of developing unique topological structures and reconfigurable materials across several length scales owing to interfacial tension and increased morphological stability on substrates^27–31^. Interestingly, molecular chirality has also been found to profoundly affect the nanostructures of interfacial assemblies of amphiphilic surfactants and rod-like viruses by the chiral control of interfacial tension^32^. Although advances have been achieved in the preparation of ring structures by the self-organization of linear polymers, discrete organic molecules and nanoparticles on solvent evaporation on a surface^33,34^, most of these interfacial assemblies are at the nanoscale and are prevailingly achiral or mixtures of conglomerates owing to insufficient hierarchical chirality control. Encouraged by our previous progress in the aqueous preparation of helicity-controlled Möbius strips with diameters of 0.5–2.5 μm (ref. ^35^), we conjecture that the re-assembly of pre-formed nanoscale molecular aggregates on a two-dimensional surface might increase the possibility of their circular stacking due to the reduced degree of freedom and chiral control of interfacial tension, leading to the formation of helical toroids with a larger length scale and favourable size dispersity.

Here we report a combined solution–interface-directed assembly approach to hierarchically construct micrometre-scale helical toroids from amphiphilic binaphthalene bisurea (BU) enantiomers on varied substrates. Morphological analyses reveal that these microtoroidal architectures are composed of helically arranged nanorod-like subunits with an average length of about 420 nm, originating from the interfacial fusion and re-assembly of intermediate solution aggregates. Our molecular dynamics (MD) simulation studies indicate that the pre-assembled molecular aggregates could act as new building blocks to further helically stack on top of each other, leading to the formation of helical microtoroids. The toroidal organization of intermediate aggregates affects the overall chiroptical properties of interfacial assemblies, supporting a microscopic-chirality-regulated structure–property relationship. We then show that the helical microtoroid can be used as a template to accommodate acceptor dye molecules. An efficient excitation energy transfer from donor BU to the chiral acceptor is thus achieved, representing a proof-of-concept light-harvesting antenna with both chirality and circularly polarized light features.

## Preparation of homochiral helical microtoroids

**Fig. 1:**
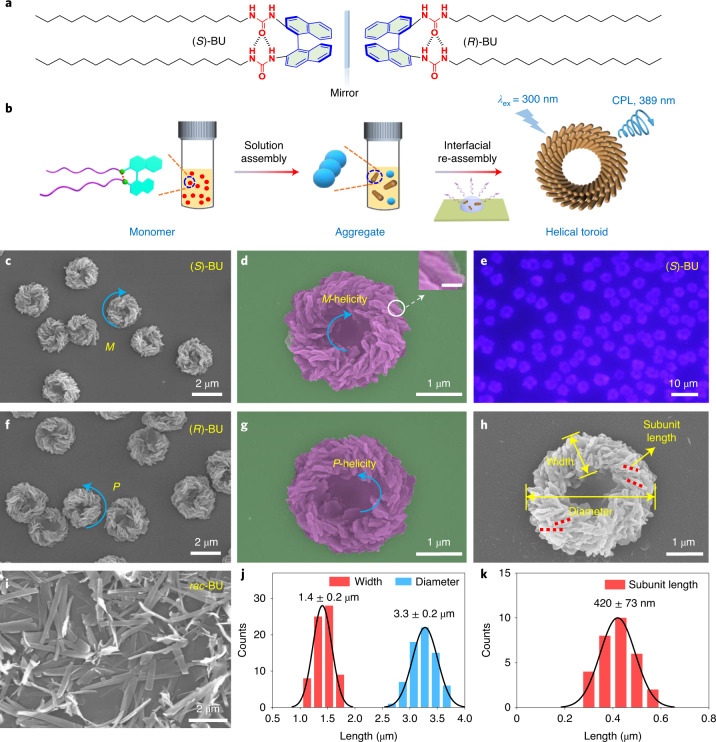
Schematic of forming helical microtoroids and morphological analysis. **a**, Chemical structures of binaphthalene BU enantiomers (*S*)-BU and (*R*)-BU, showing an intramolecular hydrogen bond between two urea groups. **b**, Illustration of the stepwise solution self-assembly of BU in supersaturated MeOH (4 mM) and interfacial re-assembly of the aggregates by natural evaporation on a surface. Expansion scale bar is 100 nm. **c**–**e**, SEM images (**c**,**d**) and fluorescence microscopy image (**e**) of (*S*)-BU interfacial assemblies on a silica wafer surface. **f**,**g**, SEM images of (*R*)-BU interfacial assemblies. **h**, Illustration of morphological parameters (diameter, width and subunit length) of an (*S*)-BU microtoroid. **i**, SEM image of interfacial assemblies from *rac*-BU. **j**,**k**, Statistical analysis of toroidal width, diameter and subunit length. The cyan arrows in **c**, **d**, **f** and **g** highlight the helicity direction of microtoroids. Here *M* and *P* represent left and right handedness, respectively. Source data

To construct helical microtoroids, a combined solution–interface-directed self-assembly strategy is proposed, which involves the stepwise self-assembly of the target compounds into molecular aggregates in solution and subsequent interfacial organization into microtoroids. For this purpose, amphiphilic binaphthalene BU enantiomers (Fig. 1a and Supplementary Scheme 1) comprising a luminescent π-chromophore, two urea moieties and two long alkyl chains were synthesized and fully characterized. These structural subcomponents encode chiroptical properties and unique self-assembly capabilities into the BU molecules, which were indirectly elaborated by a series of reference BU derivatives with alkyl chains of different lengths and methyl-substituted urea groups (Supplementary Scheme 2). BU was first investigated by two-dimensional correlation spectroscopy nuclear magnetic resonance (COSY-NMR) and selective 1D rotating-frame Overhauser effect spectroscopy (ROESY) NMR spectroscopy, and well-resolved proton signals on naphthalene were assigned (Supplementary Figs. 1 and 2). Geometry-optimized structure of (*S*)-BU by density functional theory (DFT) computation supported the existence of intramolecular hydrogen bonds between two urea groups, leading to a folded conformation of the two long alkyl chains and a binaphthalene dihedral angle of about 74.6° (Supplementary Figs. 3 and 4).

In a typical experimental protocol, the BU samples were dispersed in selected solvents, and the mixture was then heated to 343 K to afford a transparent supersaturated solution. The subsequent cooling of the BU hot solution to room temperature at a given speed (5 K min^−1^) was expected to yield intermediate molecular aggregates, which were then immediately transferred onto a two-dimensional surface via a syringe to allow their possible interfacial re-organization on ambient evaporation (Fig. 1b). Self-assembly conditions including solvent type, cooling rate and concentration were found to affect the morphologies of interfacial assemblies. Various solvents from non-polar CCl_4_ to polar dimethyl sulfoxide and protic solvents, namely, methanol (MeOH) and ethanol, were screened to evaluate the solvent effect (Supplementary Figs. 7 and 8). Micrometre-sized interfacial toroidal structures were successfully observed for MeOH, and therefore, this solvent was selected for further experimental optimizations. A proper concentration (4 mM in MeOH) was critical to form adequate interfacial BU microtoroids (Supplementary Fig. 9). The cooling rate is another important parameter in defining the supramolecular morphology as well as its functions^36^. Both slow cooling (1 K min^−1^) and fast cooling (10 K min^−1^) failed to yield helical microtoroids with satisfactory dispersity (Supplementary Figs. 10 and 11). The length of the alkyl chains and urea hydrogen bonds of the BU molecules also played important roles in controlling the interfacial self-assembled structures. Semi-spherical or spherical microstructures were obtained for BU derivatives with shortened alkyl chains ((*S*)-BU-C4, (*S*)-BU-C7 and (*S*)-BU-C12; chemical structures shown in Supplementary Scheme 2), whereas microdisc structures were formed for a BU derivative with methyl-protected urea groups ((*S*)-BU-Me; chemical structure shown in Supplementary Scheme 2), demonstrating the indispensable and collaborative roles of each subcomponent in BU molecules (Supplementary Fig. 13).

Under optimized conditions (4 mM BU in MeOH, cooling from 343 to 293 K at the speed of 5 K min^−1^), uniform-sized and large-scale microtoroids were successfully obtained on a silica wafer substrate, as observed from both scanning electron microscopy (SEM) and fluorescence microscopy images (Fig. 1c–e). A magnified SEM image of (*S*)-BU microtoroid clearly showed that the toroidal-shaped superstructure consisted of several dozens of clockwise-oriented nanorod-like subunits, showing left-handed helicity (Fig. 1d), which was further supported by transmission electron microscopy (TEM) and atomic force microscopy images (Supplementary Fig. 14). The helicity preference of these microtoroids followed the molecular chirality of BU molecules, that is, *M*-helical and *P*-helical microtoroids could be successfully obtained from (*S*)-BU and (*R*)-BU enantiomers, respectively (Fig. 1c,f and Supplementary Fig. 15). In sharp contrast, racemic BU (*rac*-BU) molecules only resulted in the formation of achiral microplates (Fig. 1i), indicating a chiral control process of interfacial re-assembly. A morphological statistical analysis gave an average outer diameter of 3.3 ± 0.2 μm and a toroidal width of 1.4 ± 0.2 μm (Fig. 1j and Supplementary Figs. 16 and 17). The average length of the helically arranged nanorod-like subunits along the microtoroidal surface was about 420 ± 73 nm (Fig. 1k and Supplementary Figs. 18 and 19). These helical microtoroidal structures could be prepared on varied substrates including silica wafer, glass, mica and quartz plate with similar outer diameters and subunit length (Supplementary Fig. 20), thus representing a universal approach for the preparation of helicity-controlled helical microtoroids.

## Characterization of molecular aggregates in solution

**Fig. 2:**
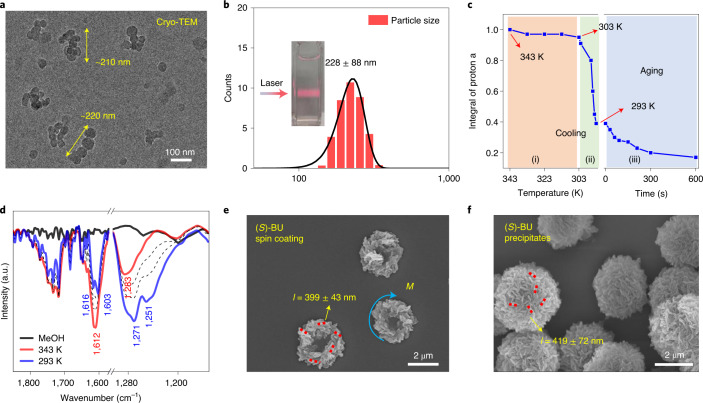
Evidence of solution aggregate formation. **a**, Cryo-TEM of vitrified MeOH solution of (*S*)-BU after cooling to 293 K. **b**, DLS data of (*S*)-BU supersaturated solution after cooling to 293 K. Inset: photograph of the Tyndall effect under a beam of red light. **c**, Plot of NMR integrals of ‘proton a’ (Supplementary Fig. 1 shows the proton assignment) against temperature and aging time obtained from variable-temperature NMR of (*S*)-BU in CD_3_OD. Sections (i)–(iii) and the corresponding colours are used to highlight three different stages. **d**, Variable-temperature FT-IR spectroscopy of (*S*)-BU solution (8 mM in MeOH). The black dashed lines are the intermediate curves between 293 and 343 K. The black solid line corresponds to the FT-IR signals of bulk MeOH at 293 K. **e**, SEM image of the spin-coated film of the solution aggregates (1,000 rpm, 60 s). **f**, SEM image of the precipitates after aging the solution aggregates over 10 min. Unless otherwise noted, the concentration of BU is 4 mM in MeOH. Source data

The formation of molecular aggregates via a solution-cooling protocol was verified by a series of experiments. The cryogenic capture of the intermediate aggregates in cooled BU MeOH solution (293 K) gave both discrete nanoparticles (diameter, ~45 nm) and predominant nanoparticle-fused aggregates (length, ~220 nm), as observed from the cryo-TEM image (Fig. 2a), indicating the colloidal nature of the supersaturated BU solution after the cooling protocol^37^. An obvious Tyndall effect (Fig. 2b, inset; Supplementary Fig. 10) and dynamic light scattering (DLS) data at 293 K also clearly supported the formation of particles with sizes of about 228 ± 88 nm (Fig. 2b), which was in the same length scale as the cryogenic aggregates but obviously shorter than the microtoroid subunits (~420 nm; Fig. 1k and Supplementary Fig. 20d,h,l). The fast spin coating of the BU intermediate colloids on a silica wafer substrate also afforded similar microtoroids with outer diameters of about 3.5 μm and periphery subunits with a length of about 399 ± 43 nm (Fig. 2e and Supplementary Figs. 21 and 22). These data demonstrated that the nanoscale aggregates underwent further morphological evolution and hierarchical self-assembly when they were transferred to the substrate. A magnified SEM image of an interfacial microtoroid clearly showed the tendency of nanoparticle fusion within a periphery subunit (Fig. 1d, inset). The cryo-TEM morphology observation indicated that the BU solution aggregates were affected by solvent types and different cooling speeds (Supplementary Figs. 23 and 24).

A spectroscopic analysis was conducted to obtain insights into the solution-phase aggregation process. Variable-temperature NMR measurements of the BU solution (4 mM in CD_3_OD) provided the in situ aggregation information. On cooling from 343 to 293 K, the chemical-shift range of aromatic protons on naphthalene became broader with ‘proton a’ and ‘proton f’ (Supplementary Fig. 26) moving downfield and upfield, respectively, demonstrating that the naphthalene rings were under the influence of both shielding and deshielding effects, which might originate from the alteration of binaphthalene dihedral angle and π–π-stacking interactions^38^. By using an internal standard method, we were able to evaluate the change in aromatic proton integrals during the aggregation process. The first stage of cooling from 343 to 303 K gave rise to slightly decreased integrals of ‘proton a’ (Fig. 2c), which demonstrated that NMR-recordable BU monomers or oligomers were the major species in this temperature range. Subsequent cooling from 303 to 293 K induced an obvious decrease in the NMR integrals owing to the formation of NMR-invisible large aggregates, which was in accordance with the cryo-TEM and DLS data. The following aging treatment of the aggregates at 293 K caused a continuous NMR integral decrease owing to further aggregation and formation of precipitates. SEM micrographs showed that these precipitates were micrometre-sized flower-like spheres consisting of several hundreds of nanoscale subunits with length of 419 ± 72 nm (Fig. 2f and Supplementary Fig. 25). Variable-temperature Fourier-transform infrared (FT-IR) spectroscopy of the BU solution showed that the stretching vibration band of the carbonyl group (*ν*-C=O at 1,612 cm^−1^) at a higher temperature (343 K) gradually split into two peaks (1,616 and 1,603 cm^−1^) on cooling to 293 K, and the bending vibration peak of the N–H bond (*δ*-N–H, 1,283 cm^−1^) was also divided into two peaks centred at 1,271 and 1,251 cm^−1^ (Fig. 2d). These splitting peaks suggested the formation of both intra- and intermolecular C=O···H–N hydrogen bonds within the solution aggregates^39,40^. All these experimental results unambiguously supported the formation of nanoscale molecular aggregates on the controlled cooling protocol of a supersaturated BU hot solution.

## Packing modes and proposed self-assembly mechanism

**Fig. 3:**
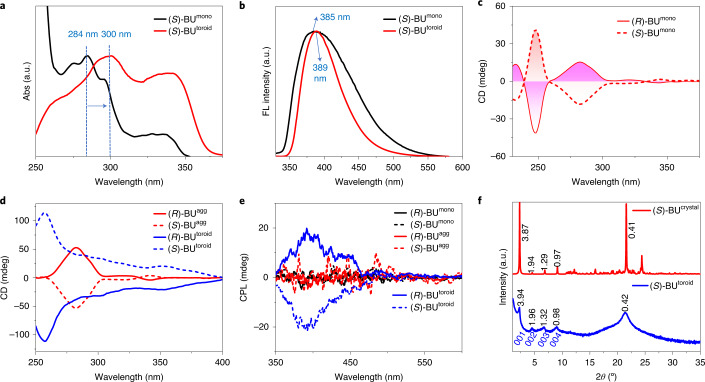
Spectroscopic analysis of hierarchical structures. **a**,**b**, UV–vis absorption (Abs) (**a**) and fluorescence (FL) (**b**) spectra of (*S*)-BU^mono^ and (*S*)-BU^toroid^. The blue arrow indicates the absorption maximum redshift of the ^1^L_a_ transition for (S)-BU^toroid^. **c**, CD spectra of (*S*)-BU^mono^ and (*R*)-BU^mono^; [BU^mono^] = 0.2 mM in MeOH, and the cuvette path length is 1 mm. **d**, CD spectra of BU^agg^ (red lines) and BU^toroid^ (blue lines); [BU^agg^] = 4 mM in MeOH, and the cuvette path length is 0.1 mm. **e**, CPL spectra of BU^mono^ (black lines), BU^agg^ (red lines) and BU^toroid^ (blue lines); the excitation wavelength is 300 nm. **f**, XRD patterns of (*S*)-BU^crystal^ (red line) and (*S*)-BU^toroid^ (blue line). Source data

A comparison of the spectroscopic characteristics of BU monomers (BU^mono^), solution aggregates (BU^agg^), interfacial microtoroids (BU^toroid^) and single-crystal structures (BU^crystal^) could bring useful information for the hierarchical self-assembly mechanism^41^. Compared with (*S*)-BU^mono^ at different concentrations (Fig. 3a and Supplementary Fig. 27), the absorption maximum of the ^1^L_a_ transition for (*S*)-BU^toroid^ was redshifted from 284 to 300 nm and the intensity of the absorption band above 300 nm was amplified (Fig. 3a), demonstrating an effective chromophore-packing arrangement within the BU interfacial toroids. The emission maximum of (*S*)-BU^toroid^ also showed a slight bathochromic shift (Fig. 3b). The chiroptical properties of hierarchical structures were studied by circular dichroism (CD) and circularly polarized luminescence (CPL) spectra. The ^1^B_b_ and ^1^L_a_ transitions of BU^mono^ showed opposite Cotton effects due to their perpendicular orientations (Fig. 3c)^42^, which was also confirmed by time-dependent DFT-calculated CD spectrum (Supplementary Fig. 5). Two enantiomers, namely, (*S*)-BU^mono^ and (*R*)-BU^mono^, exhibited mirror-imaged Cotton effects, as expected (Fig. 3c). On formation of the solution aggregates, the Cotton effect of (*S*)-BU^agg^ at the ^1^L_a_ transition (centred near 284 nm) showed a negative sign, which was in agreement with (*S*)-BU^mono^. In sharp contrast, (*S*)-BU^toroid^ gave rise to a positive Cotton effect (Fig. 3d, blue dashed line; Supplementary Fig. 28b). All the CD spectra calculated using time-dependent density functional theory for different dimeric (*S*)-BU^momo^ species showed negative Cotton effects at the ^1^L_a_ transition (Supplementary Fig. 29). Therefore, the overall positive chiroptical signals of interfacial toroids originated more from the micrometre-sized chiral structures rather than the contribution from monomeric and aggregated species. This conclusion was further unambiguously supported by the CPL spectra because both BU^mono^ and BU^agg^ showed no obvious CPL signals, whereas BU^toroid^ gave an intense CPL emission with luminescent dissymmetry factors (|*g*_lum_|) of 2 × 10^−3^ (Fig. 3e), which was due to an amplification of structural chirality within the toroidal assemblies^43^. Therefore, the toroidal organization of solution aggregates on a surface played a critical role in defining the expression of the overall chiroptical properties.

**Fig. 4:**
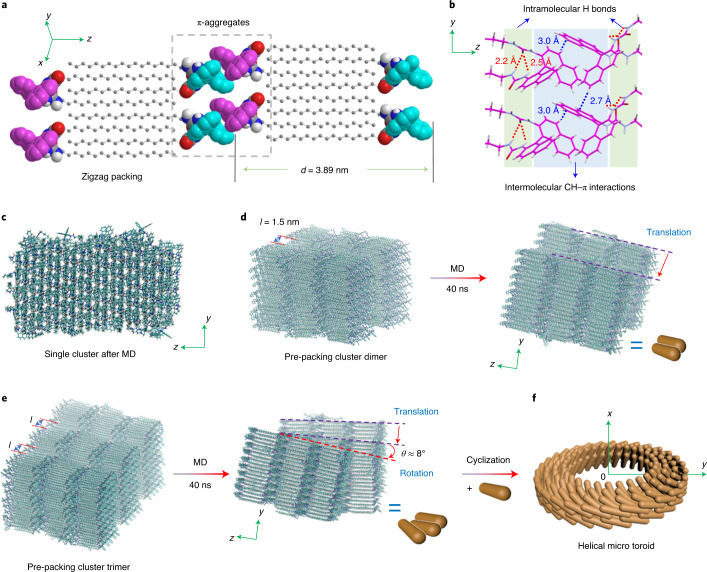
Single-crystal structures and MD simulations. **a**, Single-crystal structure of (*S*)-BU showed well-defined alkane zigzag packing and π-aggregation. **b**, Illustration of intramolecular hydrogen bonds and intermolecular CH–π interactions along two neighbouring bilayers. **c**, Equilibrium structure of a block of clusters containing 5 × 10 × 3 pairs of (*S*)-BU molecules after MD simulations. **d**, Pre-packed parallel cluster dimer shows translation along the *y* axis after 40 ns MD, where *l* is the distance between two clusters. **e**,**f**, Pre-packed parallel cluster trimer shows translation and rotation after 40 ns MD (**e**), which then undergoes further circular stacking when interacting with extra clusters (**f**).

X-ray diffraction (XRD) pattern and infrared spectrum could provide structural information for molecular packing within the assemblies. The XRD pattern of BU microtoroids showed regular diffraction peaks at 2*θ* values of 2.24°, 4.50°, 6.70° and 8.99° (Fig. 3f). The diffraction peaks calculated from the Bragg’s equation gave *d*-spacing values of 3.94 nm, 1.96 nm, 1.32 nm and 0.98 nm, which should correspond to the (001), (002), (003) and (004) diffractions, respectively (Fig. 3f, blue line), supporting a multilamellar packing mode. In the microtoroid, the CH_2_-stretching bands appeared at 2,922 cm^−1^ and 2,851 cm^−1^, thus suggesting the contributions of a gauche conformation of alkyl chains (Supplementary Fig. 30, blue line)^44^. The single-crystal structure provided direct information for the molecular packing mode in the solid state. We successfully obtained the single crystal of (*S*)-BU by a slow evaporation approach in ethyl acetate/dimethyl sulfoxide co-solvents. The single-crystal XRD data of (*S*)-BU revealed a triclinic packing diagram with a *P*1 chiral space group feature (Fig. 4a and Supplementary Table 1). The intramolecular hydrogen bonds between the two urea groups were retained in the crystal network (Fig. 4b; H···O distances, 2.2 Å and 2.5 Å). A further extension along both *y* and *z* axes was dominated by CH–π interactions (H···C distance, 2.7 Å and 3.0 Å) among neighbouring naphthalene moieties, giving a well-defined multilamellar structure (Fig. 4a; bilayer length, 3.89 nm). The XRD patterns of the single-crystal and microtoroidal assemblies of (*S*)-BU showed similar diffraction peaks, therefore proving that BU molecules in the microtoroids adopted similar packing modes to those in the solid structure. However, the diffraction peaks of (*S*)-BU^toroid^ were obviously broader than those of the single crystal, indicating that the molecular ordering within (*S*)-BU^toroid^ was not as well defined as that in the crystal, most probably due to the involvement of intermolecular hydrogen bonds, which was in accordance with the variable-temperature FT-IR spectroscopy results.

Atomistic MD simulation using the general AMBER force field (gaff2) was performed to provide insights into the possible self-assembly mechanism. The initial geometrical structure of (*S*)-BU was extracted from the single-crystal and 5 × 10 × 3 pairs of (*S*)-BU molecules were generated as one block of cluster (Fig. 4c and Supplementary Fig. 6), which was then solvated in MeOH and equilibrated through MD simulations for 40 ns after thousands of steps of energy minimization. The equilibrium configuration showed that the bilayer packing within the block almost remained, which was in agreement with the XRD pattern. Possible intercluster stacking was also simulated by placing the equilibrated cluster monomer at a distance of ~1.5 nm along the *x* axis before solvation in the MeOH solvent (Fig. 4d). The systems for the cluster dimer and trimer containing 600 and 900 (*S*)-BU molecules were solvated in more than 20,000 and 30,000 MeOH molecules with box sizes of 13.72 nm and 19.72 nm, respectively. After the energy minimization of whole systems, 40 ns MD simulations under the constant pressure/temperature (*NPT*) ensemble were performed. The cluster dimer showed relative translation along the *y* axis, whereas the cluster trimer had an obvious rotation tendency (rotation angle, about 8°) on cluster stacking on top of each other, indicating the preference of circular stacking (Fig. 4e).

Based on these experimental and computational data, we propose a possible aggregation–cyclization mechanism for the formation of interfacial microtoroids. The BU molecules in a supersaturated solution first form discrete and fused nanoparticles (aggregates) on cooling treatment, as supported by the cryo-TEM characterization data through synergistic CH–π interaction, H-bond and alkyl-chain packing. When these aggregates are placed on a substrate, solvent evaporation allows further interaggregate fusion and promotes the formation of nanoscale rod-like structures. The chiral control of interfacial tension drives the helical tilting and cyclization of nanorods, lowering the area of substrate–nanorod interface and facilitating the formation of micrometre-scale helical toroids (Fig. 4f). This mechanism was also verified by control experiments. The directional inter-nanorod interactions revealed by MD simulation should guide further self-assembly on adding extra aggregates. When transferring a second droplet of BU colloids containing molecular aggregates to cover the pre-formed microtoroid domain on silica wafer (Supplementary Fig. 31a), the toroidal topology of interfacial assemblies after natural evaporation almost remained but with more crowded subunits, leading to increased sizes of toroid diameter (Supplementary Fig. 31e). These results unambiguously indicated that the second batch of aggregates continued to assemble on the helical surface of the previous microtoroids, instead of assembling into new microtoroids, confirming effective inter-nanorod interactions.

## Energy transfer within acceptor-doped microtoroids

**Fig. 5:**
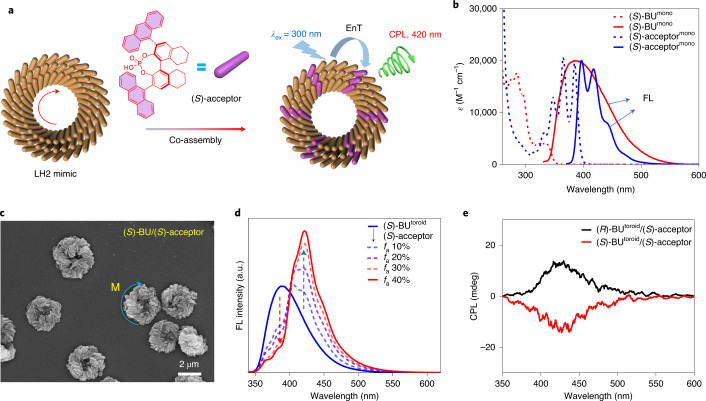
Acceptor-dye-doping experiments. **a**, Helical toroid resembles the crystal structure of LH2 and thus is used to co-assemble with a chiral acceptor dye, leading to efficient energy transfer among the two components and circularly polarized luminescence from the acceptor. EnT, energy transfer. **b**, UV–vis absorption (dashed lines) and emission spectra (solid lines) of (*S*)-BU^mono^ and (*S*)-acceptor^mono^ in dilute MeOH, showing spectral overlap between donor emission (red solid line) and acceptor absorption (blue dashed line); the emission spectra are normalized to the absorption maximum of (*S*)-acceptor^mono^ at 385 nm. **c**, SEM image of (*S*)-BU/(*S*)-acceptor interfacial microtoroids. **d**, Fluorescence spectra of (*S*)-BU/(*S*)-acceptor microtoroids in the presence of increasing (*S*)-acceptor ratios (*f*_a_). **e**, CPL spectra of (*R*)-BU/(*S*)-acceptor and (*S*)-BU/(*S*)-acceptor microtoroids (*f*_a_, 40%). For fluorescence and CPL, the excitation wavelength is 300 nm. Source data

The chiral microtoroids composed of luminescent chromophores resemble the crystal structure of natural light-harvesting antenna complex^15^. We decided to investigate whether the helical toroids can sensitize the guest dye and thus provide a platform for proof-of-concept light-harvesting antenna mimicking both chirality and circularity features (Fig. 5a)^45^. A luminescent phosphoric acid compound was screened out as the acceptor dye due to its spectral overlap and co-assembly ability with the donor BU (Fig. 5b, blue dashed line and red solid line), which enables the possibility of Förster resonance energy transfer within the co-assembled artificial antenna^46^. The addition of acceptor dye (*S*)-BU or (*R*)-BU acceptors to (*S*)-BU or (*R*)-BU donors exerted no obvious influence on the toroidal topology and helicity of nanostructures, as observed from the SEM and fluorescence microscopy images (Fig. 5c and Supplementary Figs. 33–36), indicating that the arrangement of chiral acceptor dyes followed the helical direction of BU interfacial assemblies. However, the chirality match between the acceptor and BU helical microtoroids could affect the energy transfer and chiroptical properties of their interfacial co-assemblies.

The Förster resonance energy transfer efficiency (*Ф*_ET_) in the (*S*)-BU^toroid^/(*S*)-acceptor system was slightly different from the (*R*)-BU^toroid^/(*S*)-acceptor system for acceptor loading ratios (*f*_a_) below 30% (Fig. 5d and Supplementary Fig. 37). The *Ф*_ET_ value could be improved to over 90% at a relatively high *f*_a_ value of 40% (Supplementary Fig. 38), whereas lower *f*_a_ values gave less satisfactory energy transfer efficiencies. The sign of chiroptical signals of the co-assembled antenna was defined by the helicity direction of microtoroids; thus, (*S*)-BU^toroid^/(*S*)-acceptor gave the same handedness of the CD signal as the (*S*)-BU^toroid^ (Supplementary Fig. 39, red line, and Fig. 3d, blue dashed line). The presence of a chiral acceptor dye only affected the spectral position and intensity of the Cotton effect of the diastereomeric pairs of (*S*)-BU^toroid^/(*S*)-acceptor and (*R*)-BU^toroid^/(*S*)-acceptor (Supplementary Fig. 39). When an acceptor dye with the energy match was captured by the microtoroid, Förster resonance energy transfer occurred as in many other cases^47^. However, since the system was chiral, energy-transfer-mediated CPL signal ascribed to the acceptor was successfully obtained with a |*g*_lum_| value of 2 × 10^−3^ (Fig. 5e)^48^. The structure–property relationship was further elaborated by energy transfer and CPL studies in other solvent systems. Despite the fact that energy transfer could also occur in most of the non-toroidal interfacial assemblies by using other solvents, all of them failed to give energy-transfer-related CPL signals from the acceptor dye (Supplementary Fig. 40), thus again indicating the unique role of toroidal topology in defining the expression of overall chiroptical functions.

## Conclusion

In summary, we have shown a combined solution and interface-guided chiral assembly strategy to achieve helical toroidal structures at the micrometre scale. The helicity of toroids is governed by the stereogenic configuration of BU molecules, adopting a chiral control principle of interfacial tension. Morphological analyses, spectroscopic data and computational simulations supported an aggregation–cyclization mechanism for the formation of interfacial microtoroids. The operationally simple method could afford monodispersed micrometre toroids on varied substrates due to improved morphological stability, which provides a powerful alternative approach to the limited existing methods for generating helical toroids. Furthermore, these microtoroids could emit circularly polarized luminescence and exhibit excellent host ability to accommodate guest dyes without structural deformation, in a light-harvesting toroid construct. We believe that the interfacial self-assembly protocol by drop-evaporation or fast spin-coating approaches of pre-formed solution aggregates will lead to the directed hierarchical chiral self-assembly of numerous functional colloidal dispersion systems, highlighting the prospect of larger-sized chiral superstructures with advanced topology features. These micrometre-scale structures reach a higher scale of chiral self-assembly materials compared with prevailing homochiral architectures at molecular scales or nanoscales.

## Methods

### Materials

All the commercial chemicals were used as received without further purification. The synthesis procedures of (*S*)-BU, (*R*)-BU and *rac*-BU are listed in Supplementary Scheme 1. The reference BU compounds, namely, (*S*)-BU-C4, (*S*)-BU-C7, (*S*)-BU-C11 and (*S*)-BU-Me, are listed in Supplementary Scheme 2.

### Interfacial assembly protocol

Typically, 1.75 mg (2 μmol) BU was dispersed into 0.5 ml MeOH in a 3 ml sample vial or cuvette. The mixture was heated at 343 K until the solid was entirely dissolved. The solution was cooled from 343 K to 293 K at a speed of 5 K min^−1^ with a UH4150 (Hitachi) variable-temperature accessory. A drop of the cooled solution was then transferred to the target substrate (291 K) via a syringe. After natural evaporation at room temperature (293 K), interfacial assemblies were obtained and used for further measurements.

### SEM, atomic force microscopy and TEM characterizations

SEM measurement was performed on an S4800 (Hitachi) instrument with an accelerating voltage of 10 kV and a working current of 10 µA. The atomic force microscopy image was recorded on a Dimension FastScan (Bruker Nano) instrument with a silicon tip on silicon nitride cantilevers in the tapping mode (30 μm length with typical resonant frequencies of 400 kHz and spring constant of 4 N m^−1^). TEM was performed on a JEM-1011 (JEOL) instrument with an accelerating voltage of 100 kV. Cryo-TEM was performed on a Themis300 (Thermo Scientific) instrument with an accelerating voltage of 200 kV or 300 kV.

### UV–vis spectroscopy

The solution samples were loaded in a quartz cuvette to record the UV–vis absorption spectra on a U-3900 (Hitachi) spectrophotometer. To avoid scattering effects originating from the large size of these microstructures, the solid samples were removed from the substrates with a scraper and were transferred to a BaSO_4_ sample plate, which was then measured through the UV–vis diffuse-reflectance spectrum mode on a UV-2600 (Shimadzu) spectrophotometer.

### Fluorescence spectrum

The fluorescence spectra were recorded on an F-4500 fluorescence spectrophotometer (Hitachi) at a voltage of 400 V with a 5 nm slit for both excitation and emission sides. Fluorescence decay curves were recorded on an FLS980 (Edinburgh Instruments) spectrophotometer. The fluorescence quantum yields were measured on a FluoroMax Plus (HORIBA) instrument by using an integrating sphere.

### CD spectrum

The electronic circular dichroism and linear dichroism spectra were simultaneously recorded on a CD spectrometer J-1500 (JASCO) at a scanning rate of 500 nm min^−1^ in the range of ~200–650 nm (solution, transmission mode) or ~250–650 nm (solid, diffuse-reflectance mode).

### CPL

The CPL spectra were recorded on a CPL-300 spectrophotometer (JASCO) in the range of ~350–600 nm. The excitation wavelength for all the samples was 300 nm. The *g*_lum_ spectra were transferred from the CPL spectra using the Spectra Manager software of JASCO (Version 2.12.00).

### Fluorescence microscopy image

The interfacial assemblies were observed on an IX83 (Olympus) fluorescence microscope.

### FT-IR spectrum

The ground solid samples were dispersed in a KBr pellet and submitted for FT-IR spectra measurement on Bruker Tensor 27. The liquid samples were measured on a VERTEX 70v (Bruker) FT-IR instrument.

### Powder and single-crystal XRD measurements

The solid samples were directly loaded onto a glass sample holder to record the XRD spectra on EmpyreanX (PANalytical) with Cu-Kα radiation (*λ* = 1.5406 Å) at 40 kV and 40 mA. The scanning range was from 1° to 60°. Single-crystal XRD was performed by an XtaLAB Synergy-R (Rigaku) diffractometer.

### NMR and mass spectra

Here ^1^H NMR, ^13^C NMR, COSY and ROESY spectra were measured on Bruker Avance spectrometers (Bruker BioSpin) with CD_3_OD, CDCl_3_ or acetone-d_6_ as the solvents. Matrix-assisted laser desorption/ionization Fourier-transform ion cyclotron resonance mass spectrometry was performed on an ultrafleXtreme (Bruker) mass spectrometer.

### High-performance liquid chromatography

The samples were measured on Waters 1525 binary HPLC pump with an analytical chiral column CHIRALPAK AD-H (5 µm), and an isopropanol/hexane mixture was used as the mobile phase.

### DLS

The samples were measured at 293 K by a Zetasizer Nano ZS ZEN3600 instrument (Malvern Instruments).

### DFT computation

The energy-optimized structures were obtained by DFT computation at the B3LYP 6-311 + g(d,p) level of theory. The absorption and CD spectra were calculated by time-dependent DFT methods^49^.

### MD simulations

Atomistic MD simulations have been performed in the GROMACS (version 2020.6) simulation package using gaff2. The temperature was coupled to 298 K using the Nosé–Hoover method and the pressure was coupled to 1 atm using the Parrinello–Rahman method. The cutoff scheme of 1.2 nm was implemented for the non-bonded interactions, and the particle mesh Ewald method with a Fourier spacing of 0.1 nm was applied for the long-range electrostatic interactions. All the covalent bonds with hydrogen atoms were constrained using the linear constraint solver algorithm.

### Reporting summary

Further information on research design is available in the Nature Research Reporting Summary linked to this article.

## Online content

Any methods, additional references, Nature Research reporting summaries, source data, extended data, supplementary information, acknowledgements, peer review information; details of author contributions and competing interests; and statements of data and code availability are available at 10.1038/s41565-022-01234-w.

## Supplementary information

Supplementary Information Supplementary Figs. 1–62, Schemes 1 and 2 and Table 1.

Reporting Summary

Supplementary Data 1 CheckCIF file of the (*S*)-BU crystal.

Supplementary Data 2 CheckCIF file of the *rac*-BU crystal.

## Data Availability

All relevant data are available from the corresponding authors on reasonable request. The X-ray crystallographic coordinates for structures reported in this study have been deposited at the Cambridge Crystallographic Data Centre (CCDC) under deposition numbers 2203258 for (*S*)-BU and 2203259 for *rac*-BU. These data can be obtained free of charge from the CCDC via http://www.ccdc.cam.ac.uk/data_request/cif. Source data are provided with this paper.

## Source data

Source Data Fig. 1 Statistical source data.
Source Data Fig. 2 Statistical source data.
Source Data Fig. 3 Statistical source data.
Source Data Fig. 5 Statistical source data.
