# Supplementary material for: Hierarchically self-assembled homochiral helical microtoroids
Source: Nat Nanotechnol. 2022 Nov 3;17(12):1294–302. doi: 10.1038/s41565-022-01234-w (PMC9747612; doi:10.1038/s41565-022-01234-w)
Supplement: Supplementary file 1 — Supplementary Figs. 1–62, Schemes 1 and 2 and Table 1. [file 41565_2022_1234_MOESM1_ESM.pdf]

nature nanotechnology

Article

https://doi.org/10.1038/s41565-022-01234-w

Hierarchically self-assembled homochiral helical microtoroids

In the format provided by the authors and unedited

Supplementary Information for

Hierarchically Self-Assembled Homochiral Helical Micro-Toroids

Cong Du[1,3], Zujian Li[1,3], Xuefeng Zhu[1], Guanghui Ouyang[1], and Minghua Liu[1,2]

[1]Beijing National Laboratory of Molecular Sciences and CAS Key Laboratory of Colloid, Interface and Thermodynamics, Institute of Chemistry, Chinese Academy of Sciences, North First Street 2, Zhongguancun, Beijing 100190, China.

[2]University of Chinese Academy of Sciences, No.19(A) Yuquan Road, Beijing 100049, China.

[3]These authors contributed equally.

*Correspondence to: ouyanggh@iccas.ac.cn, liumh@iccas.ac.cn.

Contents

1. Synthesis and characterization 3
2. DFT calculations and MD simulations. 8
3. Self-assembly condition optimization and morphology analysis. 10
4. Control experiments, VT-NMR and single crystal. 18
5. Acceptor dye doping experiments. 25
6. Additional spectra. 29
7. Single crystal data 42

1. Synthesis and characterization

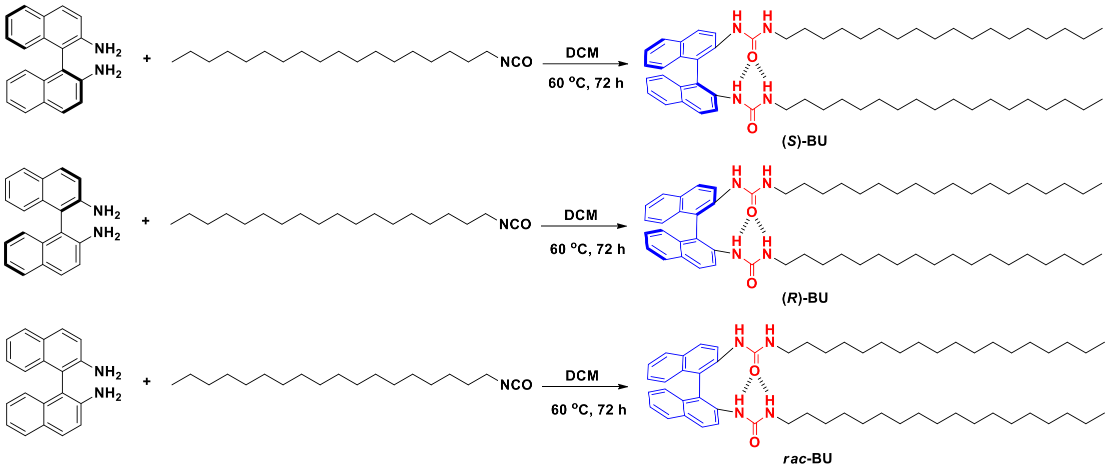

Scheme S1. Synthesis routes to (*S*)-BU, (*R*)-BU and *rac*-BU.

Compound (*S*)-BU: (*S*)-(-)-1,1'-Binaphthyl-2,2'-diamine (568 mg, 2.0 mmol), octadecyl isocyanate (1733 mg, 6.0 mmol) were added in a three necked, round-bottomed flask under argon atmosphere. DCM (50 mL) was injected *via* a syringe into the flask. The mixture was refluxed at 60 °C for 72 h. After cooling to room temperature, the reaction mixture was concentrated in vacuum. The residue was purified by column chromatography (silica gel, eluent: n-hexane/ethyl acetate = 5:1, v/v). The product was obtained as a white powder, 1.17 g, yield, 67 %.

HR-MALDI-TOF-MS: Calc.: $C_{58}H_{90}N_4O_2$ = 874.7064, found: 875.7128 $[M+H]^+$, 897.6948 $[M + Na]^+$.

^{1}H NMR (500 MHz, CD_3OD, ppm): δ 8.27 (d, *J* = 9.1 Hz, 2H), 8.03 (d, *J* = 9.1 Hz, 2H), 7.94 (d, *J* = 8.2 Hz, 2H), 7.39 (t, *J* = 7.5 Hz, 2H), 7.23 (t, *J* = 7.7 Hz, 2H), 6.92 (d, *J* = 8.5 Hz, 2H), 3.14-2.94 (m, 4H), 1.36-1.21 (m, 64H), 0.92 (t, *J* = 6.8 Hz, 6H).

^{13}C NMR (100 MHz, $CDCl_3$, ppm): δ 156.17, 135.83, 132.86, 130.76, 129.54, 128.14, 127.01, 125.21, 125.01, 122.26, 120.70, 40.53, 31.93, 29.71, 29.68, 29.66, 29.62, 29.55, 29.36, 29.26, 26.77, 22.68, 14.10.

Elementary analysis: Calc. for (*S*)-BU, [$C_{58}H_{90}N_4O_2$]: C, 79.58; H, 10.36; N, 6.40. Found: C, 79.11; H, 10.30; N, 6.46.

Melting point: 46-47 °C.

HPLC retention time: The fraction appeared at 3.96 min on an analytical chiral column CHIRALPAK AD-H, 5um, eluent was isopropanol/hexane, v/v=1:25, flow rate was 0.78 mL/min, 293 K.

Compound (*R*)-BU was synthesized as the same procedures as (*S*)-BU. White powder, yield, 69%.

HR-MALDI-TOF-MS: Calc.: $C_{58}H_{90}N_4O_2$ = 874.7064, found: 875.7128 $[M+H]^+$, 897.6948 $[M + Na]^+$.

^{1}H NMR (400 MHz, CD_3OD, ppm): δ 8.26 (d, *J* = 9.1 Hz, 2H), 8.03 (d, *J* = 9.1 Hz, 2H), 7.94 (d, *J* = 8.3 Hz, 2H), 7.39 (t, *J* = 7.1 Hz, 2H), 7.23 (t, *J* = 7,1 Hz, 2H), 6.93 (d, *J* = 8.6 Hz, 2H), 3.11-2.97 (m, 4H), 1.37-1.20 (m, 64H), 0.92 (t, *J* = 6.7 Hz, 6H).

^{13}C NMR (100 MHz, $CDCl_3$, ppm): δ 155.96, 136.23, 132.92, 130.56, 129.46, 128.11, 126.90, 125.21, 124.80, 121.99, 119.76, 40.45, 31.93, 29.84, 29.72, 29.68, 29.66, 29.63, 29.57, 29.36, 29.28, 26.82, 22.69, 14.10.

Elementary analysis: Calc. for (*R*)-BU, [$C_{58}H_{90}N_4O_2$]: C, 79.58; H, 10.36; N, 6.40. Found: C, 79.03; H, 10.21; N, 6.51.

Melting point: 46-47 °C.

HPLC retention time: The faction appeared at 5.558 min on an analytical chiral column CHIRALPAK AD-H, 5um, eluent was isopropanol/hexane, v/v=1:25, flow rate was 0.78 mL/min, 293 K.

Compound *rac*-BU was synthesized as the same procedures as (*S*)-BU. 1.31g, yield, 75 %.

HR-MALDI-TOF-MS: Calc.: $C_{58}H_{90}N_4O_2$ = 874.7064, found: 875.7133 $[M+H]^+$, 897.6954 $[M + Na]^+$.

^{1}H NMR (300 MHz, $CDCl_3$, ppm) δ 8.29 (d, *J* = 8.9 Hz, 2H), 7.96 (d, *J* = 9.0 Hz, 2H), 7.87 (d, *J* = 8.1 Hz, 2H), 7.38 (t, *J* = 7.4 Hz, 2H), 7.20 (t, *J* = 7.7 Hz, 2H), 6.97 (d, *J* = 8.5 Hz, 2H), 6.25 (s, 2H), 2.96 (s, 4H), 1.32-1.15 (m, 64H), 0.88 (t, *J* = 6.4 Hz, 6H).

^{13}C NMR (100 MHz, $CDCl_3$, ppm): δ 155.78, 136.29, 132.93, 130.61, 129.59, 128.16, 126.95, 125.24, 124.81, 121.80, 119.39, 40.53, 31.95, 29.89, 29.73, 29.69, 29.63, 29.57, 29.37, 29.29, 26.83, 22.70, 14.11.

Elementary analysis: Calc. for *rac*-BU, [$C_{58}H_{90}N_4O_2$]: C, 79.58; H, 10.36; N, 6.40. Found: C, 78.94; H, 10.36; N, 6.30.

Melting point: 166-167 °C.

HPLC retention time: The first fraction appeared at 3.924 min, the second fraction appeared at 5.754 min on an analytical chiral column CHIRALPAK AD-H, 5um, eluent was isopropanol/hexane, v/v=1:25, flow rate was 0.78 mL/min, 293 K.

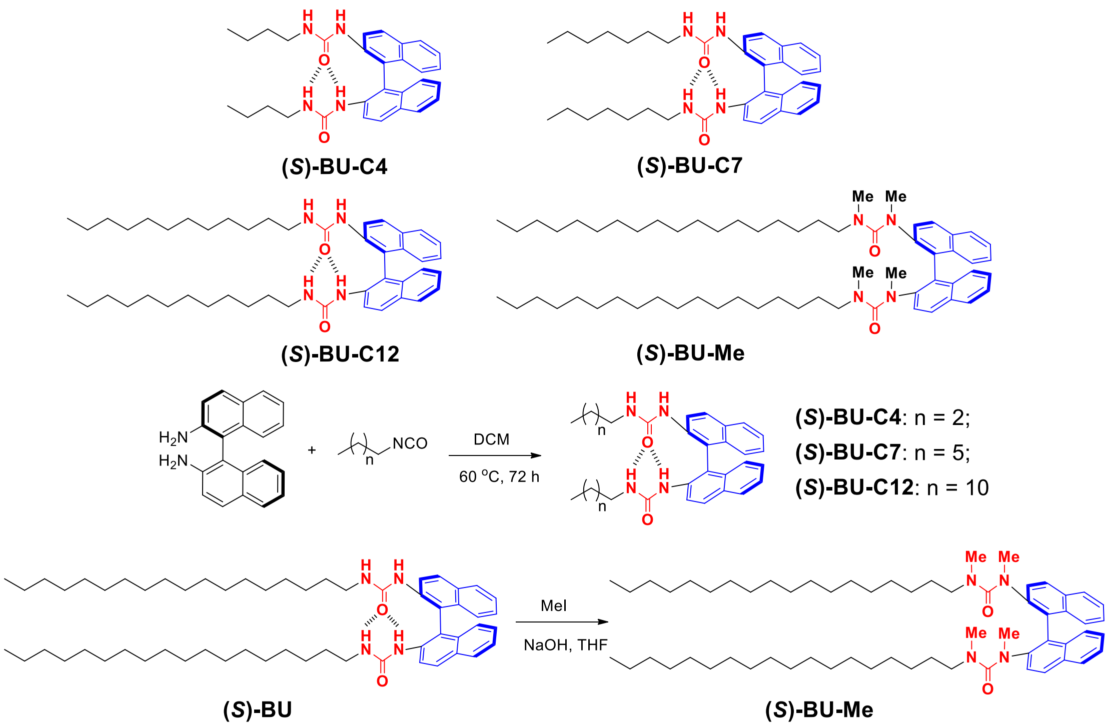

Scheme S2. Chemical structures and synthetic routes of reference BU compounds.

The synthetic procedures of reference compounds (*S*)-BU-C4, (*S*)-BU-C7 and (*S*)-BU-C12 are the same to that of (*S*)-BU.

Compound (*S*)-BU-C4: white powder, 607.3 mg, yield, 62 %.

HR-MALDI-TOF-MS: Calc.: $C_{30}H_{34}N_4O_2$ = 482.2682, found: 483.2752 $[M+H]^+$, 505.2572 $[M + Na]^+$.

^{1}H NMR (400 MHz, $CDCl_3$, ppm): δ 8.36 (d, *J* = 9.1 Hz, 2H), 7.98 (d, *J* = 9.1 Hz, 2H), 7.89 (d, *J* = 8.2 Hz, 2H), 7.39 (t, *J* = 7.6 Hz, 2H), 7.22 (t, *J* = 7.6 Hz, 2H), 6.99 (d, *J* = 8.5 Hz, 2H), 6.09 (s, 2H), 4.58 (s, 2H), 3.01 (s, 4H), 1.33-1.18 (m, 8H), 0.83 (t, *J* = 7.3 Hz, 6H).

^{13}C NMR (100 MHz, $CDCl_3$, ppm): δ 155.92, 136.28, 132.94, 130.60, 129.52, 128.14, 126.93, 125.23, 124.81, 121.92, 119.61, 40.14, 31.92, 19.91, 13.66.

Compound (*S*)-BU-C7: white powder, 646.2 mg, yield, 57 %.

HR-MALDI-TOF-MS: Calc.: $C_{36}H_{46}N_4O_2$ = 566.3621, found: 567.3690 $[M+H]^+$, 589.3510 $[M + Na]^+$.

^{1}H NMR (400 MHz, $CDCl_3$, ppm): δ 8.36 (d, *J* = 9.0 Hz, 2H), 7.99 (d, *J* = 9.0 Hz, 2H), 7.89 (d, *J* = 8.2 Hz, 2H), 7.39 (t, *J* = 7.5 Hz, 2H), 7.21 (t, *J* = 7.4 Hz, 2H), 6.99 (d, *J* = 8.4 Hz, 2H), 6.09 (s, 2H), 4.57 (s, 2H), 3.00 (s, 4H), 1.36-1.15 (m, 20H), 0.91-0.79 (m, 6H).

^{13}C NMR (100 MHz, $CDCl_3$, ppm): δ 155.84, 136.27, 132.91, 130.55, 129.53, 128.14,

126.92, 125.22, 124.79, 121.84, 119.44, 40.47, 31.73, 29.85, 28.91, 26.76, 22.58, 14.05.

Compound (*S*)-BU-C12: white powder, 904.9 mg, yield, 64 %.

HR-MALDI-TOF-MS: Calc.: $C_{46}H_{66}N_4O_2$ = 706.5186, found: 707.5253 $[M+H]^+$, 729.5073 $[M + Na]^+$.

^{1}H NMR (300 MHz, $CDCl_3$, ppm): δ 8.32 (d, *J* = 9.0 Hz, 2H), 7.96 (d, *J* = 9.0 Hz, 2H), 7.87 (d, *J* = 8.1 Hz, 2H), 7.37 (t, *J* = 7.1 Hz, 2H), 7.19 (t, *J* = 7.6 Hz, 2H), 6.96 (d, *J* = 8.4 Hz, 2H), 6.17 (s, 2H), 4.69 (s, 2H), 2.96 (d, *J* = 5.0 Hz, 4H), 1.37-1.06 (m, 40H), 0.88 (t, *J* = 7.0 Hz, 6H).

^{13}C NMR (75 MHz, $CDCl_3$, ppm): δ 155.81, 136.24, 132.90, 130.57, 129.55, 128.13, 126.90, 125.24, 124.79, 121.84, 119.52, 40.49, 31.93, 29.87, 29.67, 29.65, 29.61, 29.56, 29.36, 29.28, 26.82, 22.69, 14.11.

Compound (*S*)-BU-Me: (*S*)-BU (438 mg, 0.5 mmol), NaOH (400 mg, 10.0 mmol) were added in a three necked, round-bottomed flask under argon atmosphere. THF (50 mL), MeI (710 mg, 5.0 mmol) were injected *via* a syringe into the flask. The mixture was stirred at room temperature for 7 d. After the reaction was complete, the reaction mixture was concentrated by using rotary evaporator. The residue was purified by column chromatography (silica gel, eluent: dichloromethane/acetone = 10: 1). The product was obtained as a white powder, 195.6 mg, yield, 42 %.

HR-MALDI-TOF-MS: Calc.: $C_{62}H_{98}N_4O_2$ = 930.7690, found: 931.7742 $[M+H]^+$, 953.7562 $[M + Na]^+$.

^{1}H NMR (400 MHz, Acetone-D_6, ppm): δ 8.02 (d, *J* = 8.8 Hz, 2H), 7.95 (d, *J* = 8.2 Hz, 2H), 7.57 (d, *J* = 8.8 Hz, 2H), 7.41 (t, *J* = 7.5 Hz, 2H), 7.23 (t, *J* = 7.7 Hz, 2H), 7.05 (d, *J* = 8.6 Hz, 2H), 3.14 (s, 6H), 2.45 (s, 4H), 1.88 (s, 6H), 1.33-0.99 (m, 64H), 0.88 (t, *J* = 6.7 Hz, 6H).

^{13}C NMR (101 MHz, $CDCl_3$, ppm) δ 161.16, 134.35, 131.09, 129.72, 128.17, 127.56, 127.01, 125.08, 124.76, 49.89, 34.55, 31.94, 29.72, 29.67, 29.63, 29.57, 29.37, 27.10, 26.83, 22.70, 14.12.

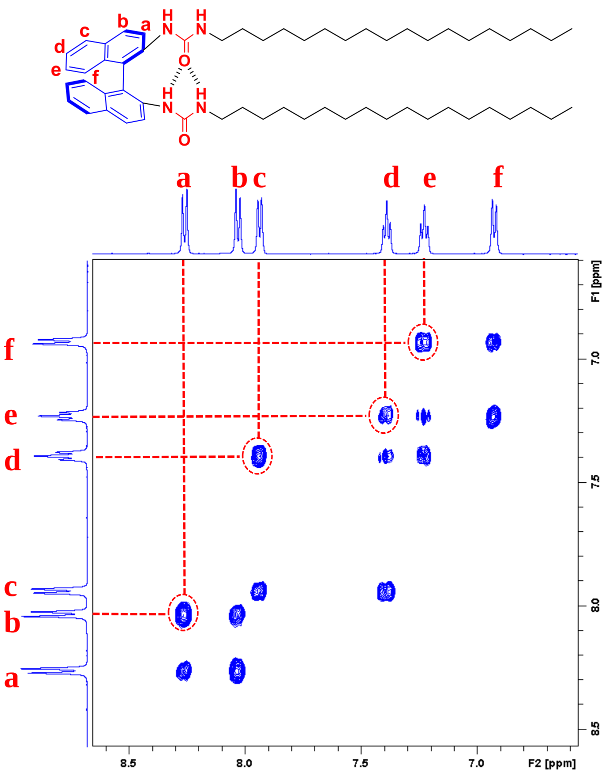

Figure S1. 2D-COSY NMR of (*S*)-BU (500 MHz, 293 K, CD_3OD). Concentration 2 mM. The letter sequences of **a-f** label the aromatic hydrogens on naphthalene.

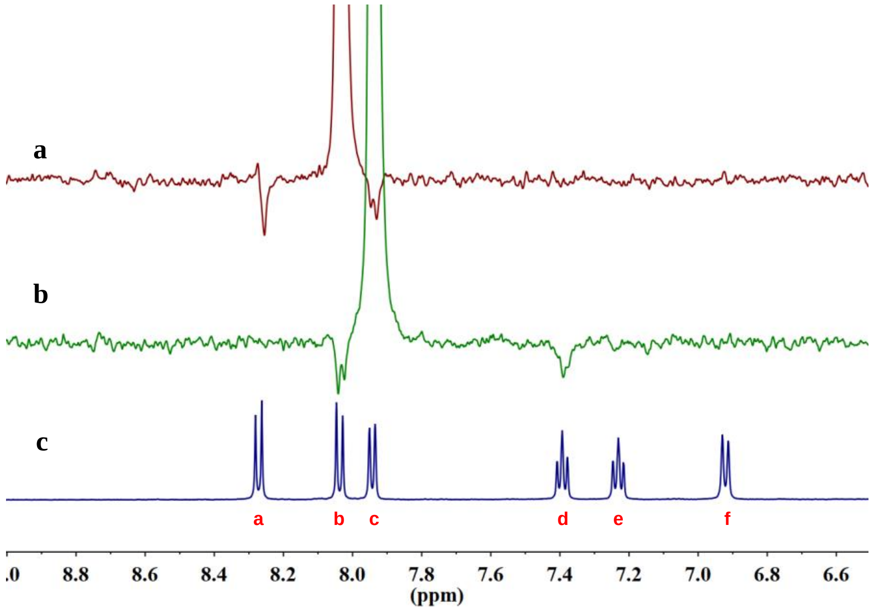

Figure S2. Selective 1D ROESY NMR spectra of (*S*)-BU (500 MHz, 293 K, CD_3OD) with selective excitation on **a**, hydrogen b and **b**, hydrogen c. **c**, the normal ^1H NMR spectrum (500 MHz, 293 K, CD_3OD) was used for comparison. The selective 1D ROESY experiments permit to obtain NOE effects between neighboring hydrogen atoms when excitation with selected resonance.

2. DFT calculations and MD simulations.

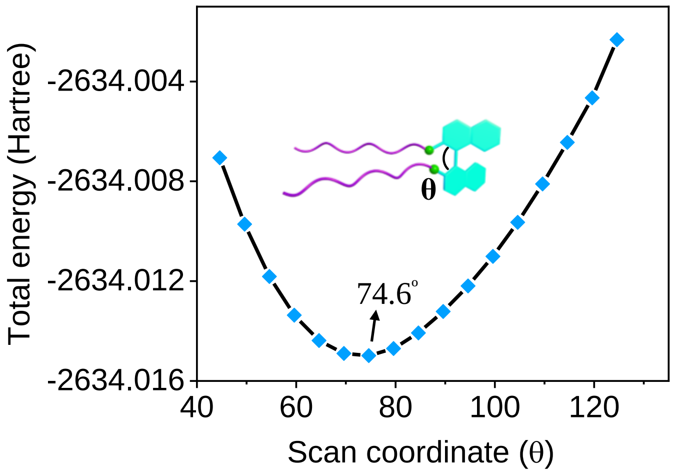

Figure S3. Scan of total energy by changing binaphthalene dihedral angle (θ). Calculated by DFT at B3LYP 6-311 G(d) level.

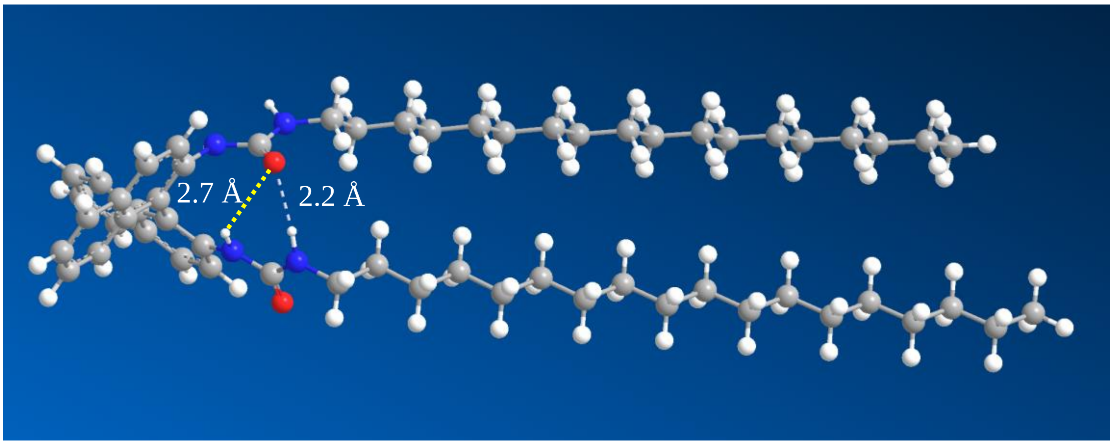

Figure S4. Energy-minimum optimized structure of (*S*)-BU by DFT computation at B3LYP 6-311 G(d,p) level. The solid balls represent: gray (carbon), white (hydrogen), blue (nitrogen) and red (oxygen). The white dashed line (O···H distance 2.2 Å) indicates intramolecular hydrogen bond, while the yellow dashed line shows that the other O···H length (2.7 Å) is out of effective H-bond distance.

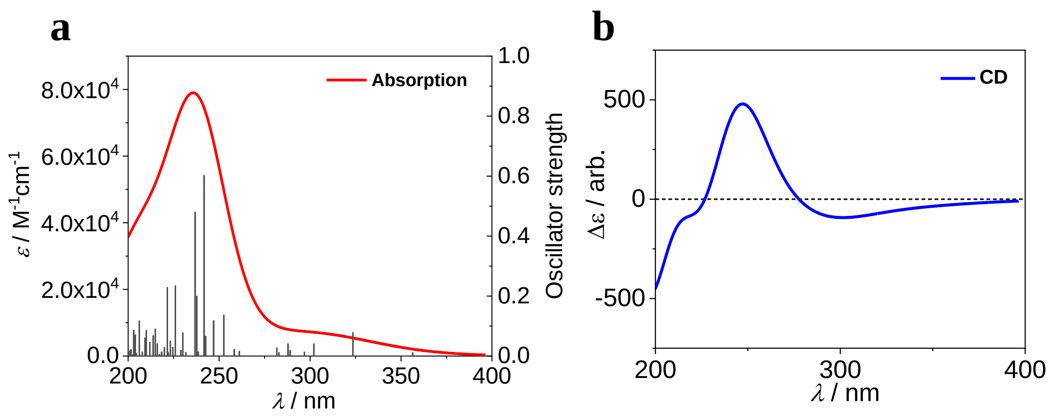

Figure S5. a, Calculated UV-vis absorption and **b**, CD spectra of (*S*)-BU by TD-DFT method at B3LYP 6-311 G(d,p) level.

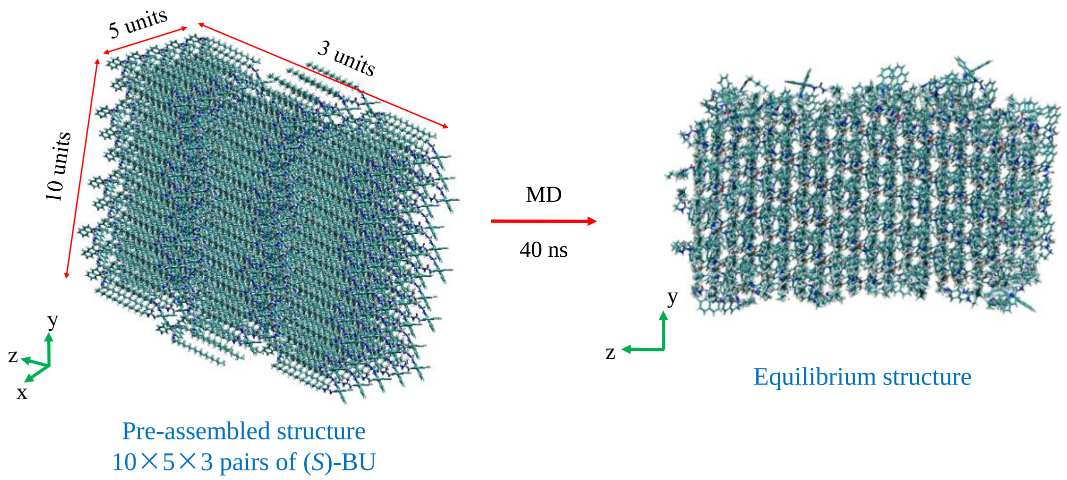

Figure S6. Molecular dynamic simulation of a pre-assembled structure containing 5×10×3 pairs of (*S*)-BU molecules. The initial geometrical BU structure is extracted from its single crystal structure.

3. Self-assembly condition optimization and morphology analysis.

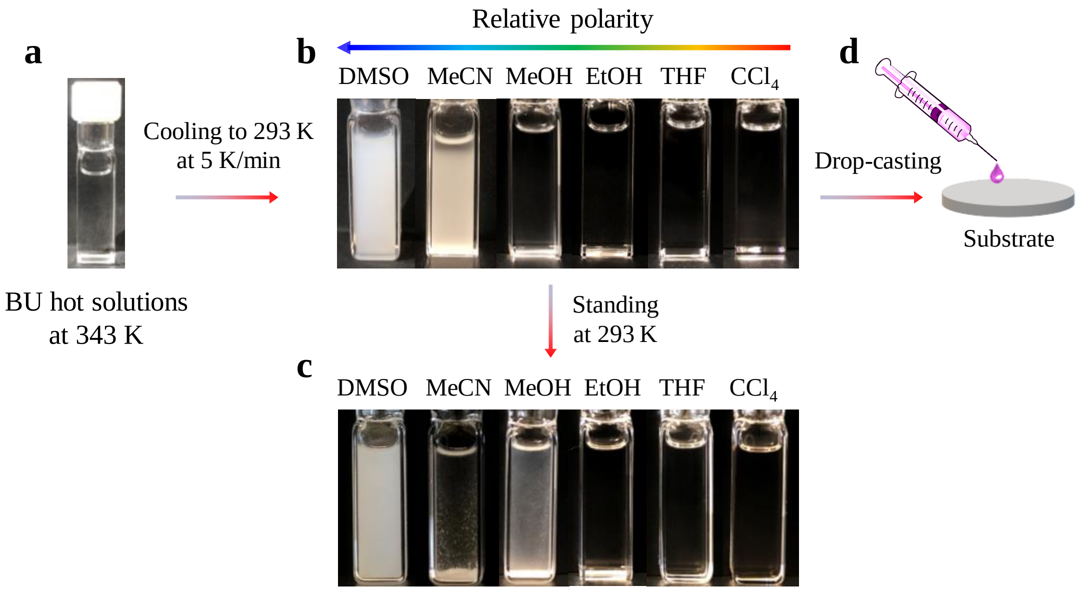

Figure S7. Solution self-assembly in different solvents. **a**, A representative photograph of a cuvette containing hot BU solutions. **b**, Cuvette photographs after cooling the hot BU solutions to 293 K at a speed of 5 K/min in different solvents. **c**, Cuvette photographs after standing over 10 min at 293 K. **d**, Schematic illustration of drop-casting the cooled BU solutions on a silica wafer substrate. [BU] = 4 mM. The bright cuvette bottom was caused by ambient light reflection when taking the photographs.

Upon cooling to 293 K, dispersions were obtained in DMSO and acetonitrile (MeCN), while other solvents (MeOH, EtOH, CCl_4 and tetrahydrofuran, THF) kept as the transparent solutions (Fig. S7b). After standing over 10 min at 293 K, the BU MeOH solution gradually underwent precipitation, while the other BU solutions (EtOH, CCl_4 and THF) were still transparent (Fig. S7c), indicating good solubility of BU compounds in these solvents. Drop-casting all the cooled solutions onto silica wafer substrates led to the emergence of different interfacial structures (Fig. S8), revealing an obvious solvent effect in controlling interfacial self-assembly morphologies.

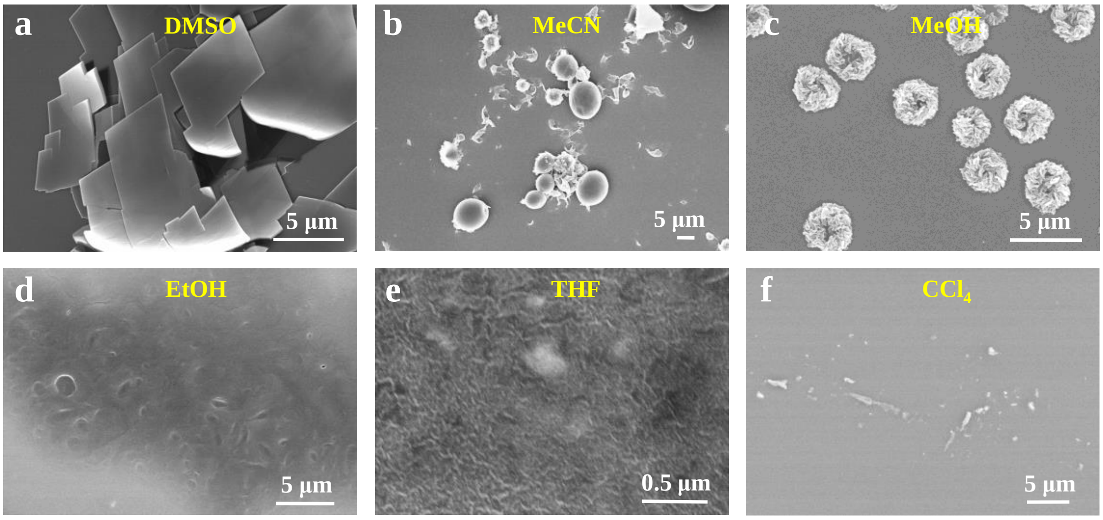

Figure S8. SEM images of (*S*)-BU interfacial assemblies on silica wafer substrate obtained in different solvents, **a**, DMSO. **b**, MeCN. **c**, MeOH. **d**, EtOH. **e**, THF. **f**, CCl_4. Cooling from 343 to 293 K at a speed of 5 K/min, then transferring the cooled solution to substrate. [(*S*)-BU] = 4 mM.

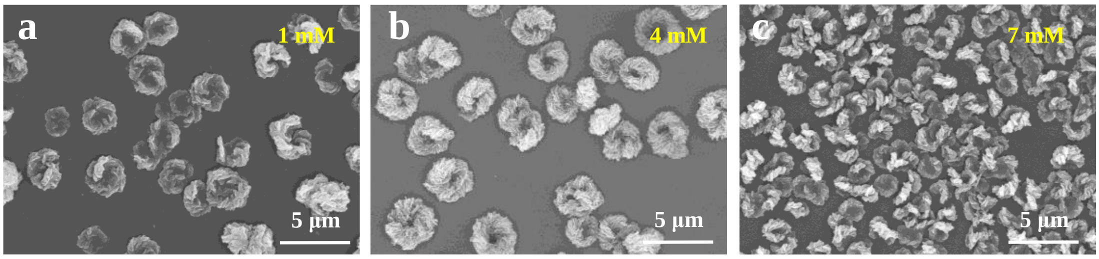

Figure S9. SEM images of (*S*)-BU interfacial assemblies on silica wafer substrate at different concentrations in MeOH. **a**, 1 mM. **b**, 4 mM. **c**, 7 mM. Cooling from 343 to 293 K at a speed of 5 K/min, then transferring the cooled solution to substrate. Lower (1 mM) and higher (7 mM) concentrations led to the formation of small amount of micro-toroids and helical structures with flat bottom in the core region, respectively.

The cooled MeOH solution by different cooling speed showed distinct Tyndall effect (Fig. S10), which should be caused by the formation of solution aggregates with different sizes. Slow cooling at 1 K/min afforded irregular precipitates (S11a), demonstrating the thermodynamic instability of the intermediate aggregates. Cooling of BU solution at 5 K/min obtained helical micro-toroids with satisfied dispersity (Fig. S11b). Fast cooling at 10 K/min gave micro-structures with obvious flat bottom in the core region (Fig. S11c), most likely due to the presence of sufficient BU monomers, which were incapable of forming solution aggregates due to fast cooling. This hypothesis was verified by the interfacial pan-like structures obtained from transferring the hot BU solution (343 K) containing exclusive monomers to silica wafer substrate (Fig. S12a).

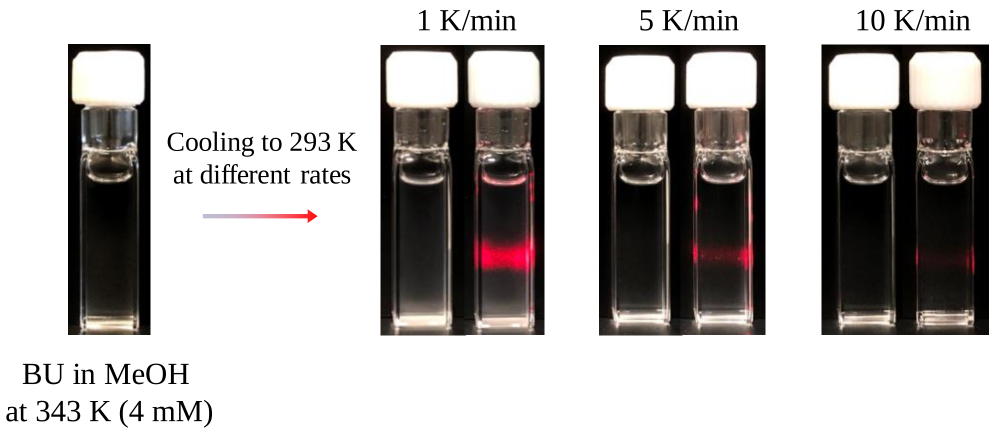

Figure S10. Cuvette photographs of BU methanol solutions after cooling to 293 K at different rates. The right photographs in each panel showed the Tyndall effects when a beam of light from a red laser diode passed through the cuvette samples. The bright cuvette bottom was caused by ambient light reflection when taking the photographs. [(*S*)-BU] = 4 mM.

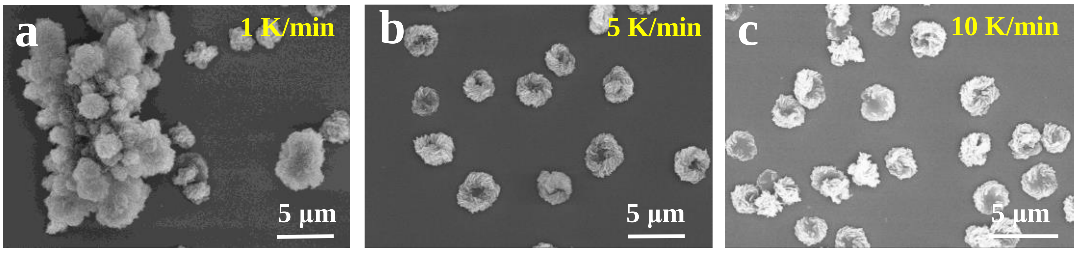

Figure S11. SEM images of (*S*)-BU interfacial assemblies on silica wafer substrate obtained at different cooling rates from 343 to 293 K. **a**, 1 K/min. **b**, 5 K/min. **c**, 10 K/min. [(*S*)-BU] = 4 mM in MeOH.

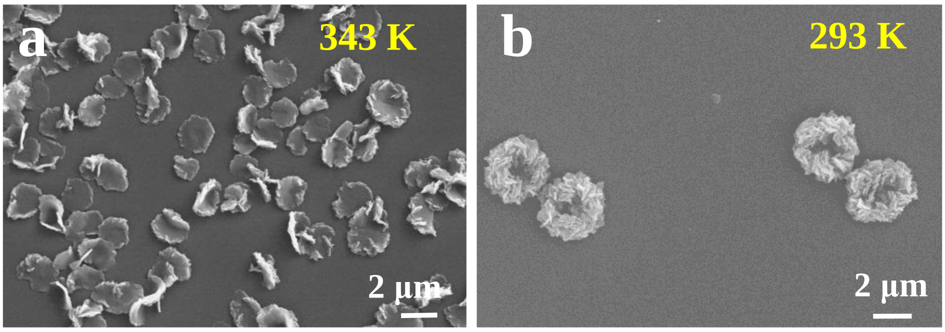

Figure S12. a, SEM image of (*S*)-BU interfacial assemblies obtained by transferring the hot solution (343 K) onto silica wafer substrate. **b**, SEM image of (*S*)-BU interfacial assemblies obtained by transferring the cooled solution (293 K) after cooling from 343 to 293 K at a speed of 5 K/min. [(*S*)-BU] = 4 mM in MeOH.

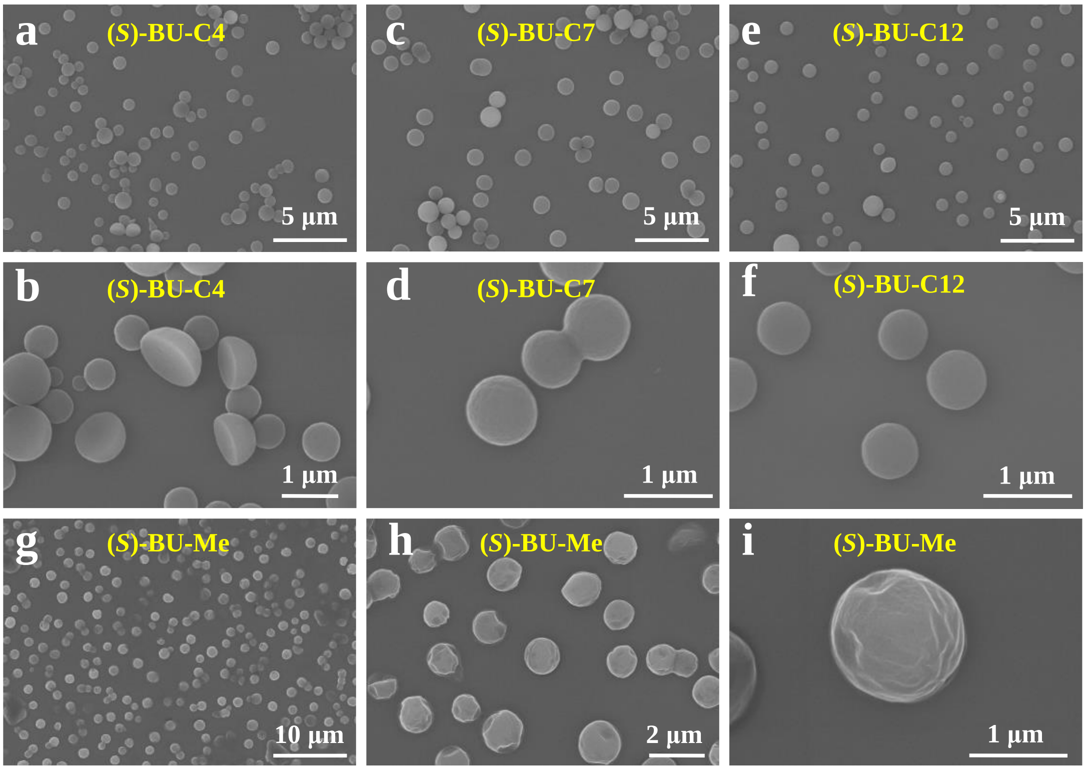

Figure S13. SEM images of reference compounds under the same self-assembly protocol to (*S*)-BU. **a**-**b**, (*S*)-BU-C4. **c**-**d**, (*S*)-BU-C7. **e**-**f**, (*S*)-BU-C12. **g**-**i**, (*S*)-BU-Me.

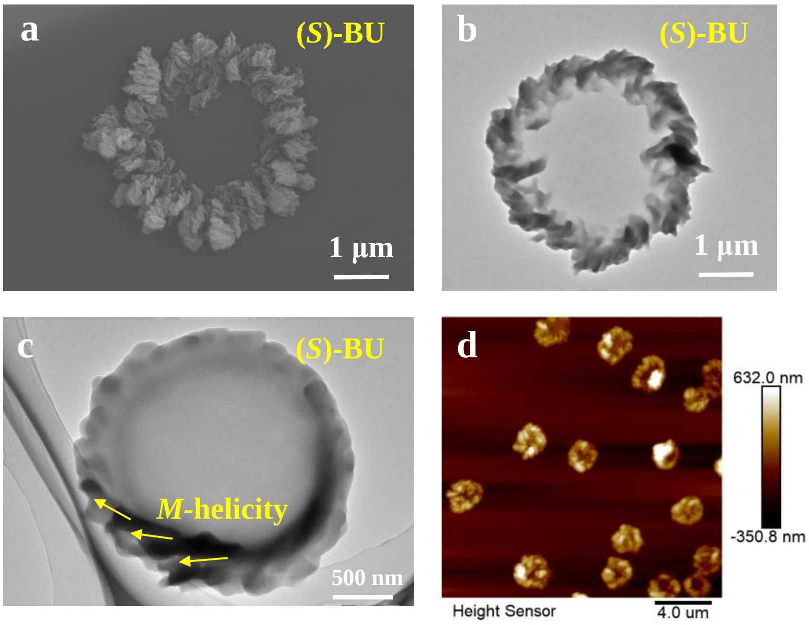

Figure S14. **a**, SEM and **b**-**c**, TEM images of (*S*)-BU interfacial assemblies on carbon-coated copper grid substrate. **d**, AFM image of (*S*)-BU interfacial assemblies on mica substrate.

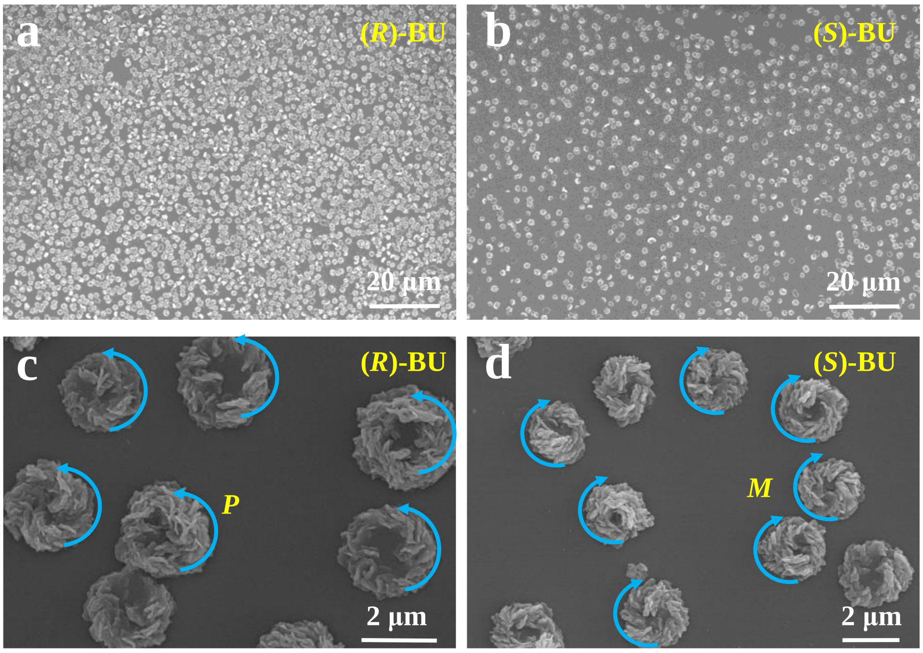

Figure S15. SEM images of **a** and **c**, (*R*)-BU micro-toroids, **b** and **d**, (*S*)-BU micro-toroids on silica wafer substrate.

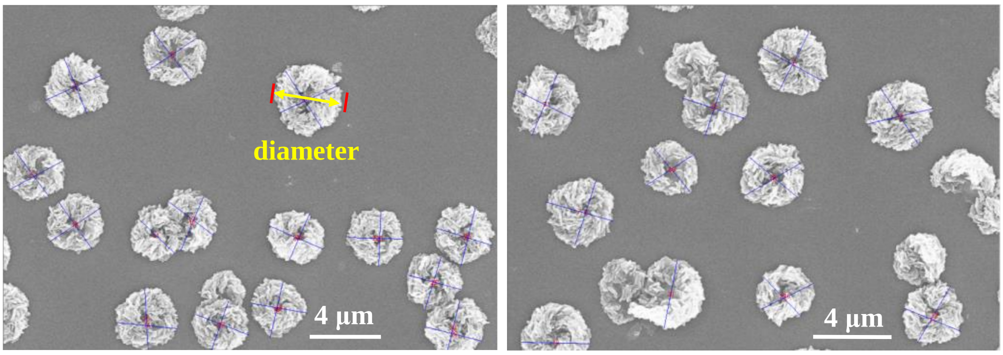

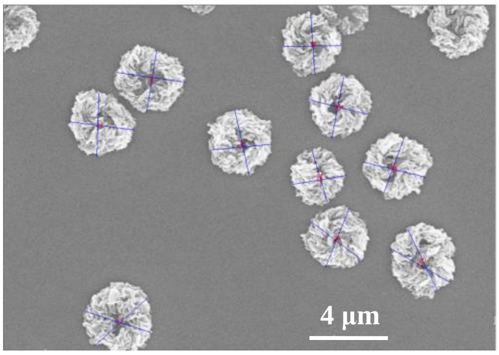

Section	Counts	Percentage
2.7-2.9 µm	5	7.2 %
2.9-3.1 µm	9	13.0 %
3.1-3.3 µm	20	29.0 %
3.3-3.5 µm	23	33 .3 %
3.5-3.7 µm	11	15.9 %
3.7-3.9 µm	1	1 .4 %

Figure S16. Statistical analysis of toroidal diameter. Each micro-toroid is measured two times. The length (blue lines) are measured with a Nano measurer software (version 1.2). The bottom right table summarizes the distributions of toroidal diameter.

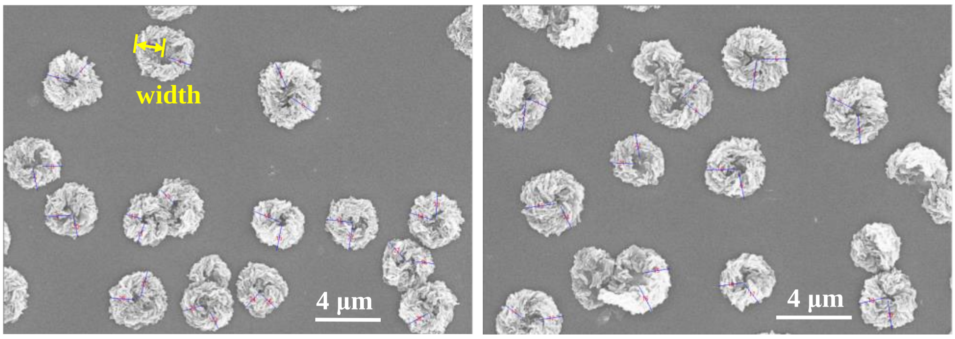

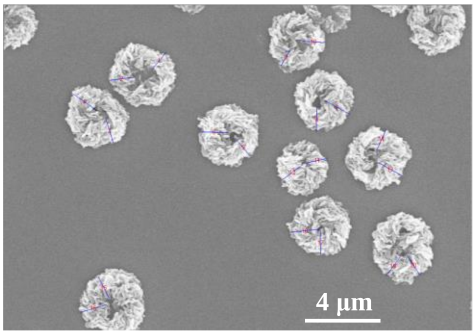

Section	Counts	Percentage
1.0-1.2 μm	8	11.4 %
1.2-1.4 μm	25	35.7 %
1.4-1.6 μm	28	40.0 %
1.6-1.8 μm	9	12.9 %

Figure S17. Statistical analysis of toroidal width. The length (blue lines) are measured with a Nano measurer software (version 1.2). The bottom right table summarizes the distributions.

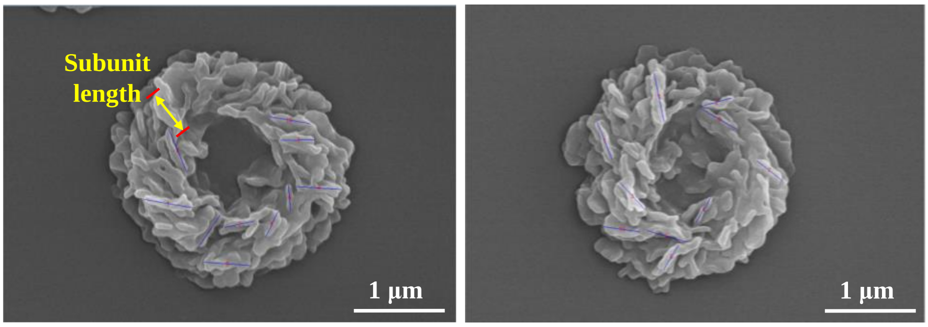

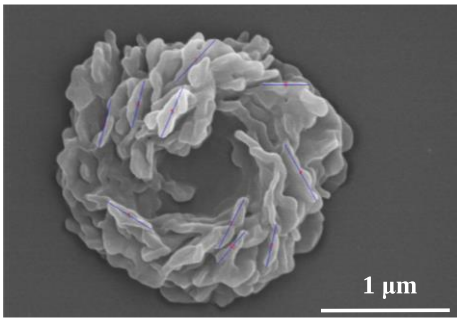

Section	Counts	Percentage
0.27-0.33 μm	4	13.3 %
0.33-0.40 μm	8	26.7 %
0.40-0.47 μm	10	33.3 %
0.47-0.53 μm	6	20.0 %
0.53-0.60 μm	2	6.7 %

Figure S18. Statistical analysis of subunit length along the (*S*)-BU micro-toroidal surface. The length (blue lines) are measured with a Nano measurer software (version 1.2). The bottom right table summarizes the distributions.

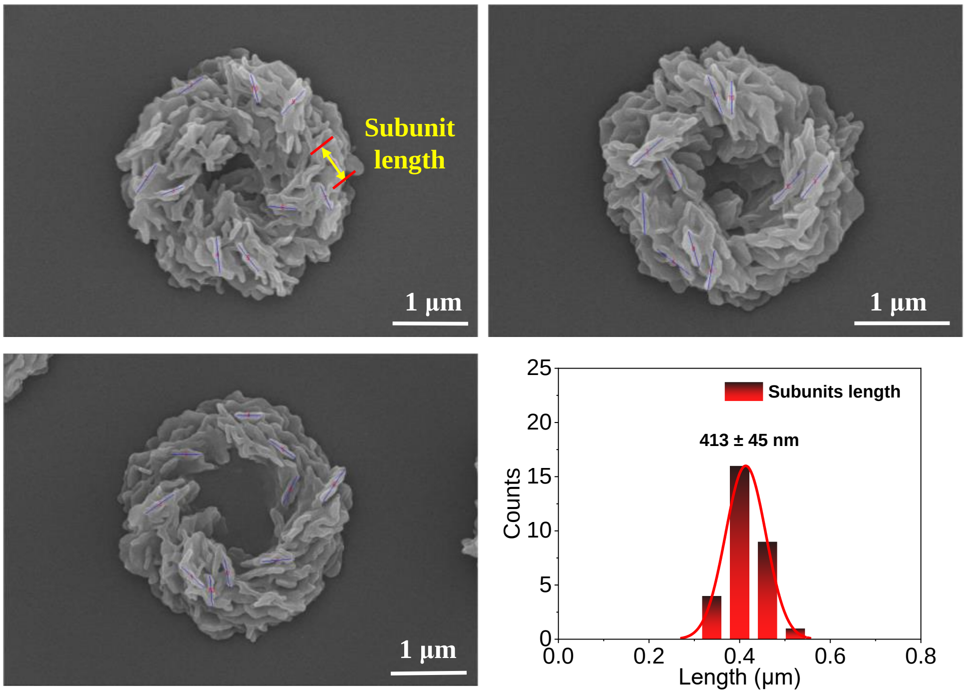

Figure S19. Statistical analysis of subunit length along the (*R*)-BU micro-toroidal surface. The length (blue lines) are measured with a Nano measurer software (version 1.2).

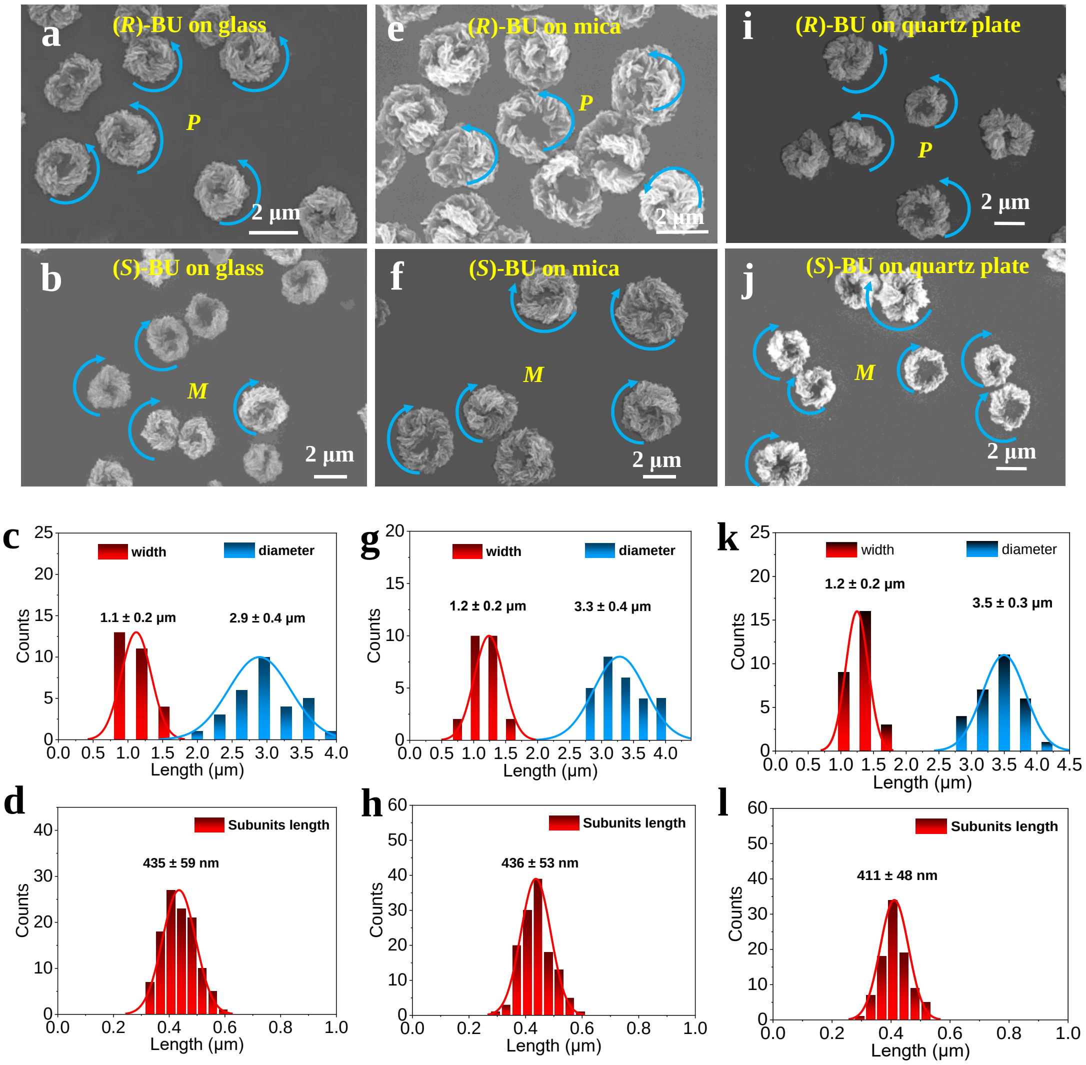

Figure S20. a-d, SEM images of (*R*)-BU and (*S*)-BU on glass substrate and their morphology parameters. **e-h**, SEM images of (*R*)-BU and (*S*)-BU on mica substrate and their morphology parameters. **i-l**, SEM images of (*R*)-BU and (*S*)-BU on quartz plate substrate and their morphology parameters.

4. Control experiments, VT-NMR and single crystal.

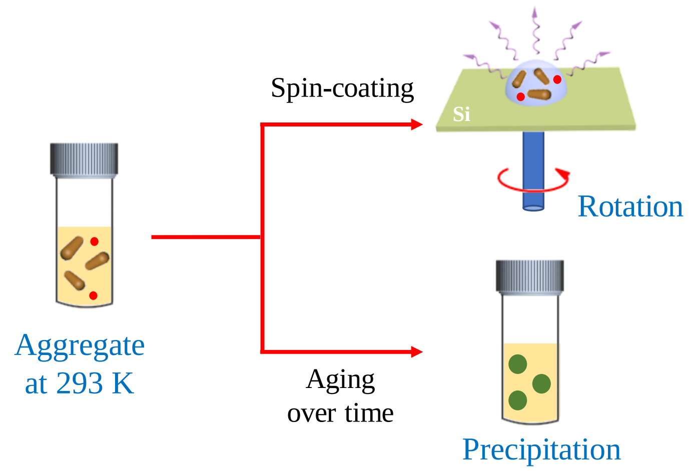

Figure S21. Illustration of spin-coating and aging treatments of the pre-formed solution aggregates. [(*S*)-BU] = 4 mM in MeOH.

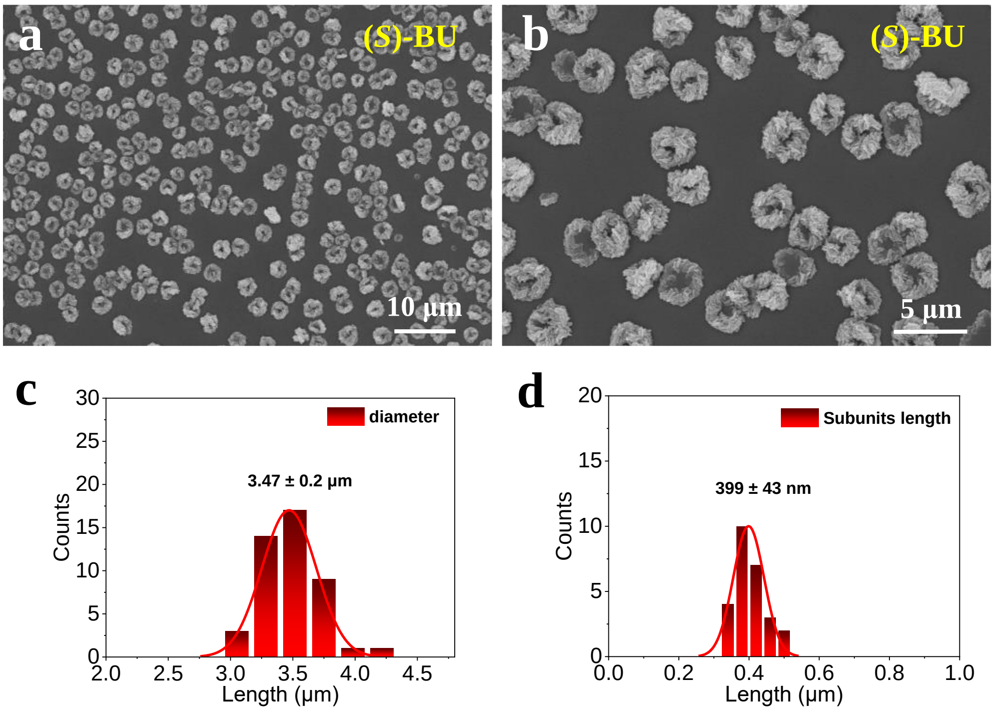

Figure S22. a-b, SEM images of (*S*)-BU interfacial assemblies by fast spin-coating of the cooled (*S*)-BU solution (293 K) on a silica wafer substrate. 1000 rpm for 60 s. **c-d**, Statistical analysis of the toroidal diameter and subunits length with spin-coating protocol.

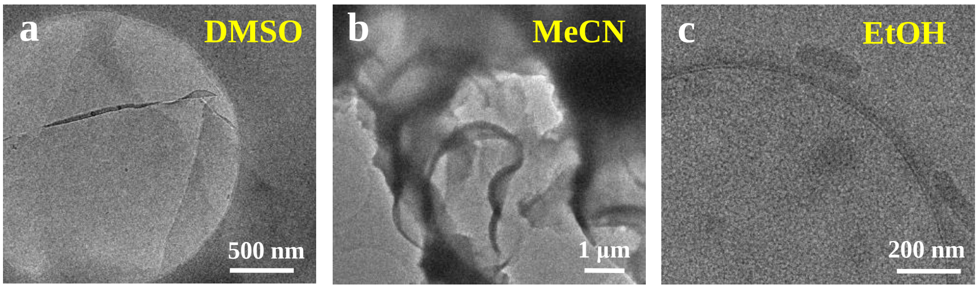

Figure S23. Cryo-TEM images of vitrified BU samples in different solvents. **a**, DMSO. **b**, MeCN. **c**, EtOH. [BU] = 4 mM.

Micro-sheet structures were observed in DMSO and MeCN and nano-sized plates in EtOH (Fig. S23), while no obvious aggregation structures could be obtained in other solvents. Slow cooling at 1 K/min led to the formation of precipitates and fast cooling at 10 K/min failed to form nanoscale aggregates as demonstrated by distinct Tyndall effects (Fig. S10) and cryo-TEM micrographs (Fig. S24).

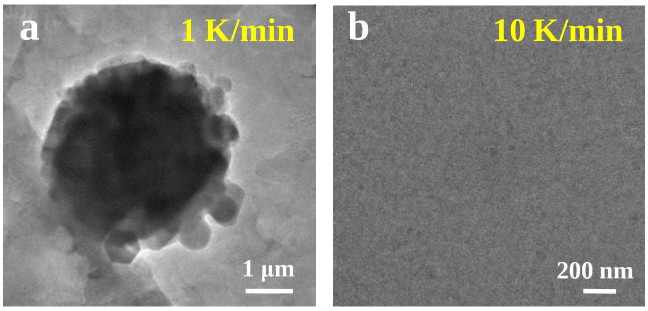

Figure S24. Cryo-TEM images of vitrified MeOH solution of BU samples after cooling to 293 K at different rates. **a**, 1 K/min. **b**, 10 K/min. [BU] = 4 mM.

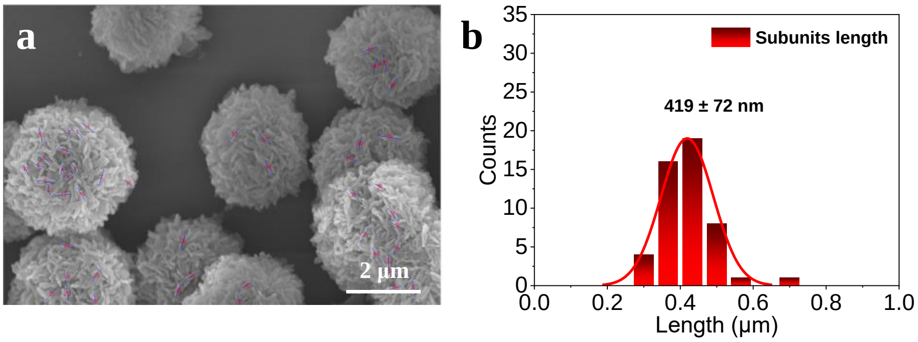

Figure S25. a, SEM image and **b**, Statistical analysis of the subunits length of (*S*)-BU precipitates in Fig. 2f of the main text.

After cooling from 343 to 293 K, the (*S*)-BU colloids were allowed to stand for 10 min, and naked-eye visible precipitates could be observed in the cuvette. SEM image indicated that these precipitates were composed of micro-spheres. Their subunits have similar length scale of about 419±72 nm to those measured in micro-toroids.

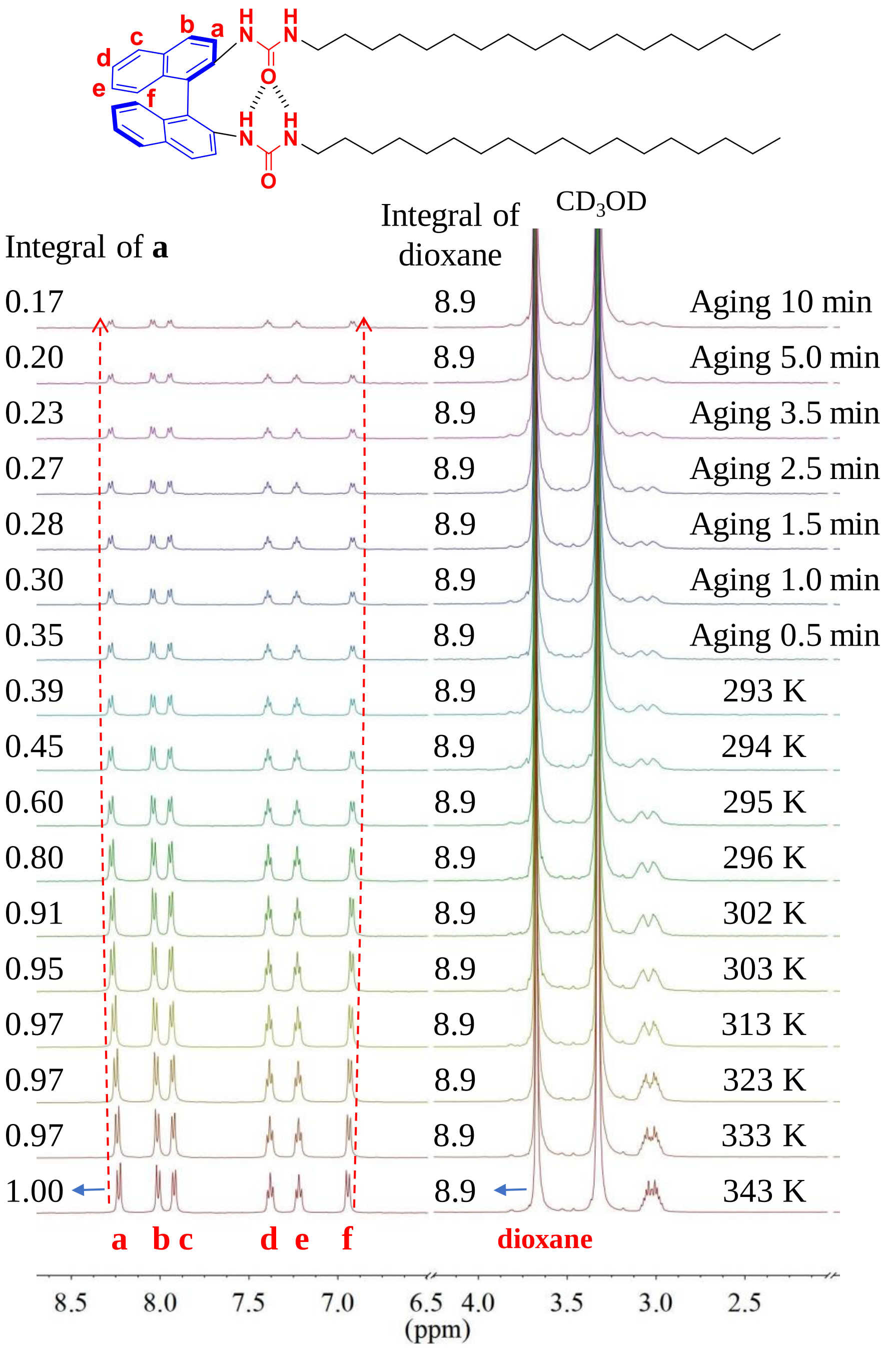

Figure S26. Variable-temperature NMR spectra of (*S*)-BU in CD_3OD (4 mM). Dioxane was added as an internal standard to evaluate the NMR integral change of aromatic protons on naphthalene during the solution self-assembly process. (*S*)-BU/dioxane = 1 : 2.225 in molar ratio. After cooling to 293 K, the NMR sample was hold at this temperature for 10 min to evaluate further precipitation.

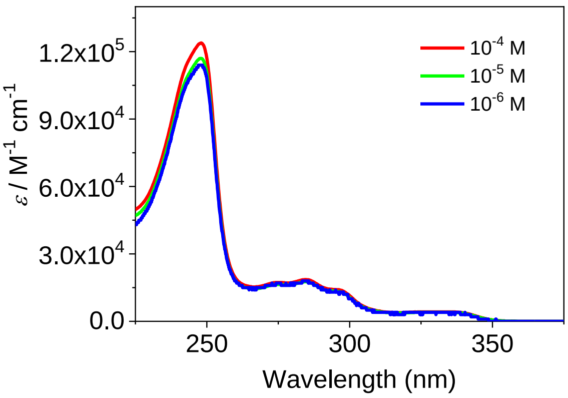

Figure S27. Concentration-dependent UV-vis absorption spectra of (*S*)-BU in dilute MeOH. 293 K.

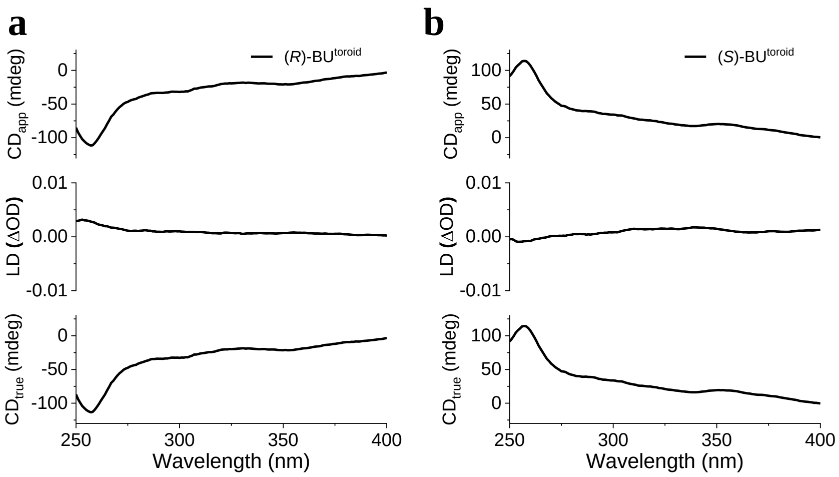

Figure S28. The apparent CD (upper), LD (middle) and true CD (bottom) signals of BU micro-toroids. It could be found that the contribution of LD to the true CD signals of all the samples was negligible.

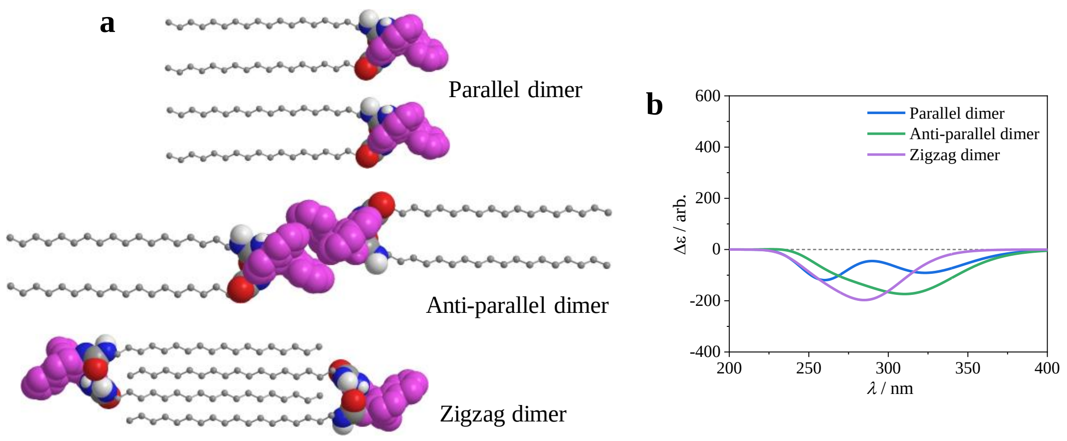

Figure S29. a, Representative geometries of dimeric (*S*)-BU species, the structures are extracted from the BU single crystal. **b**, Calculated CD spectra of different dimers in **a** by TD-DFT method.

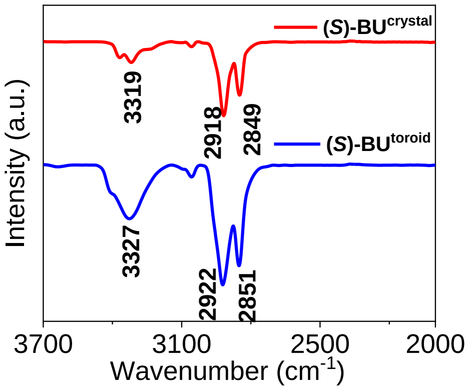

Figure S30. FT-IR spectra of (*S*)-BU^{crystal} (red line) and (*S*)-BU^{toroid} (blue line).

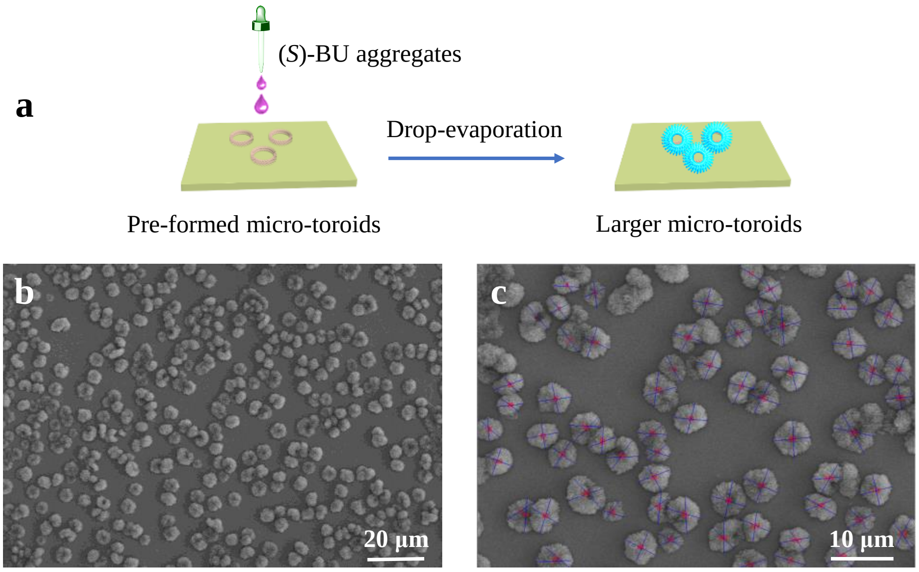

d

Section	Counts	Percentage
2.5-3 µm	2	1.6 %
3-3.5 µm	12	9.7 %
3.5-4 µm	21	16.9 %
4-4.5 µm	38	30.7 %
4.5-5 µm	25	20.2 %
5-5.5 µm	20	16.1 %
5.5-6 µm	4	3.2 %
6-6.5 µm	2	1.6 %

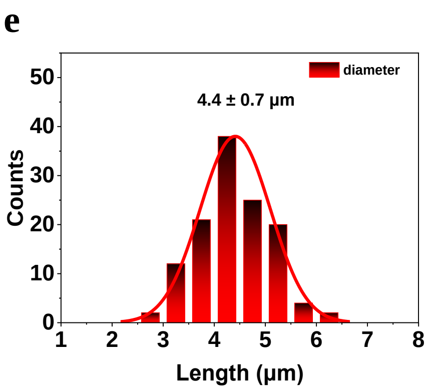

Figure S31. **a**, Schematic representation of covering the pre-formed micro-toroids by adding a second droplet of (S)-BU colloids containing aggregates. **b-c**, SEM images of (*S*)-BU micro-toroids by second-droplet method. **d-e**, Statistical analysis of toroidal diameter.

a

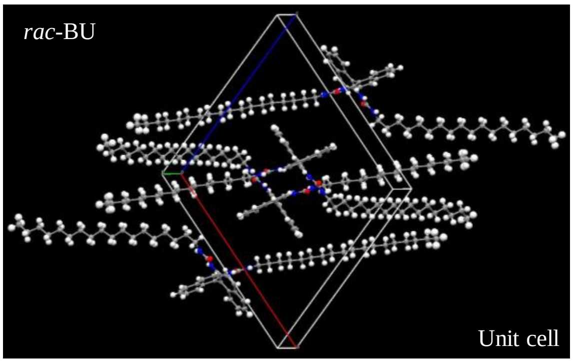

b

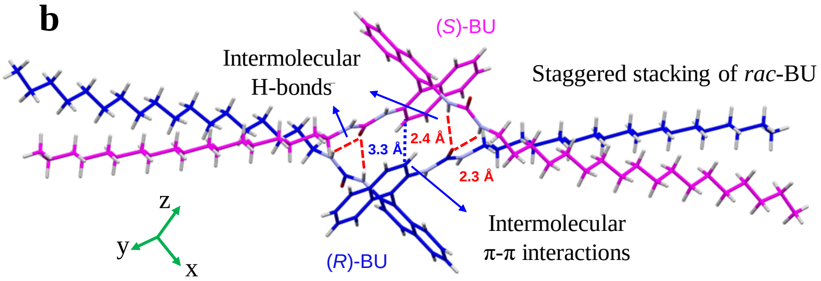

Figure S32. a, The unit cell of single crystal structure of *rac*-BU. **b**, X-ray crystallography analysis showed that a pair of (*R*)- and (*S*)-BU enantiomers were hold together by multiple intermolecular hydrogen bonds among urea groups.

5. Acceptor dye doping experiments.

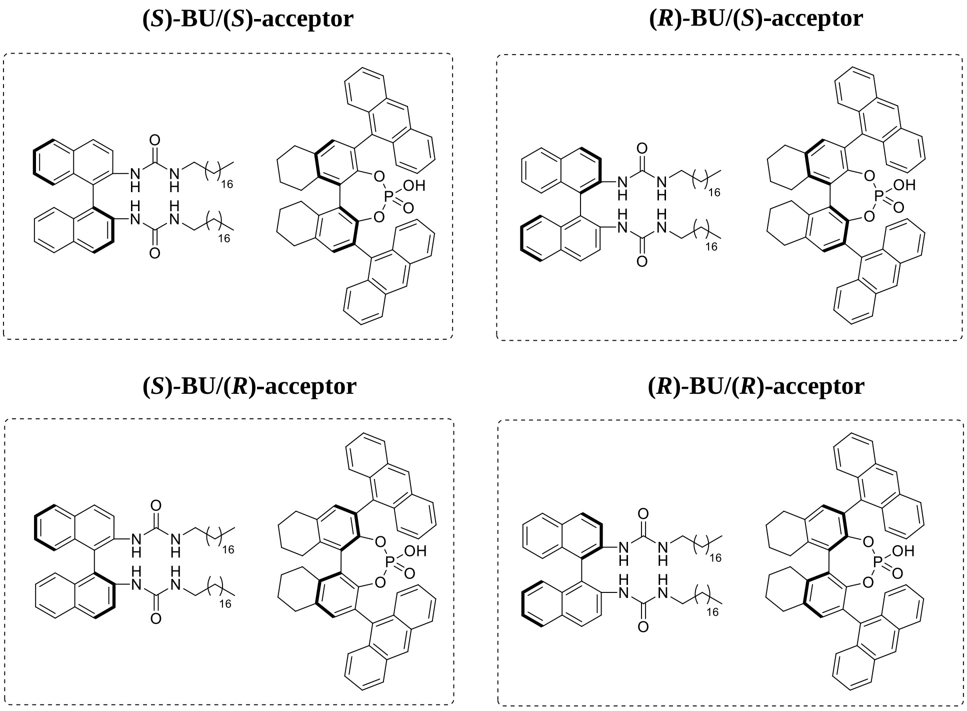

Figure S33. Chemical structures of BU and acceptor dye and four possible chiral combination.

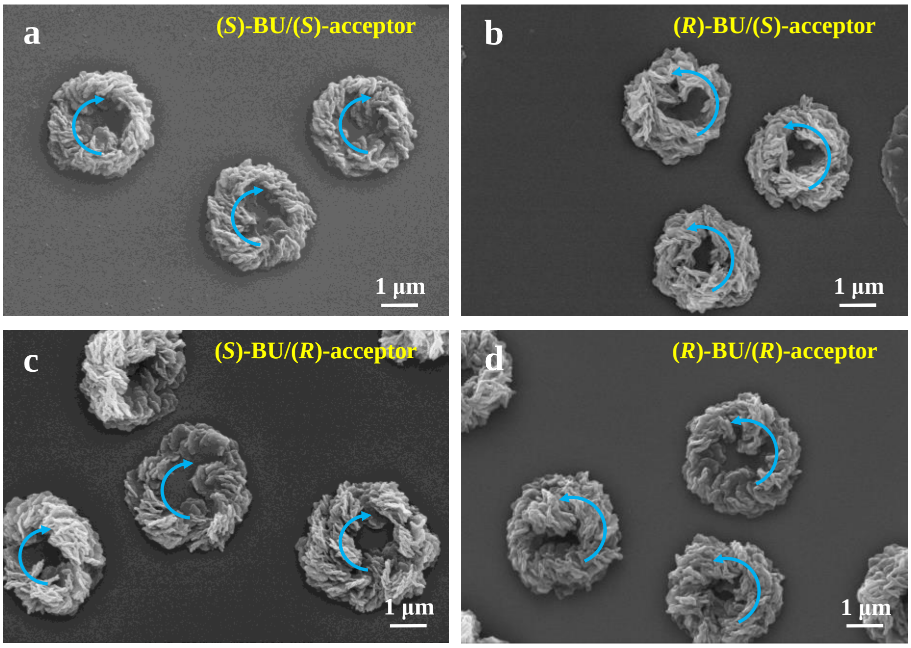

Figure S34. SEM images of different combination co-assemblies. **a**, (*S*)-BU/(*S*)-acceptor. **b**, (*R*)-BU/(*S*)-acceptor. **c**, (*S*)-BU/(*R*)-acceptor. **d**, (*R*)-BU/(*R*)-acceptor. Acceptor molar ratio is 40 %. [BU] = 4 mM in methanol.

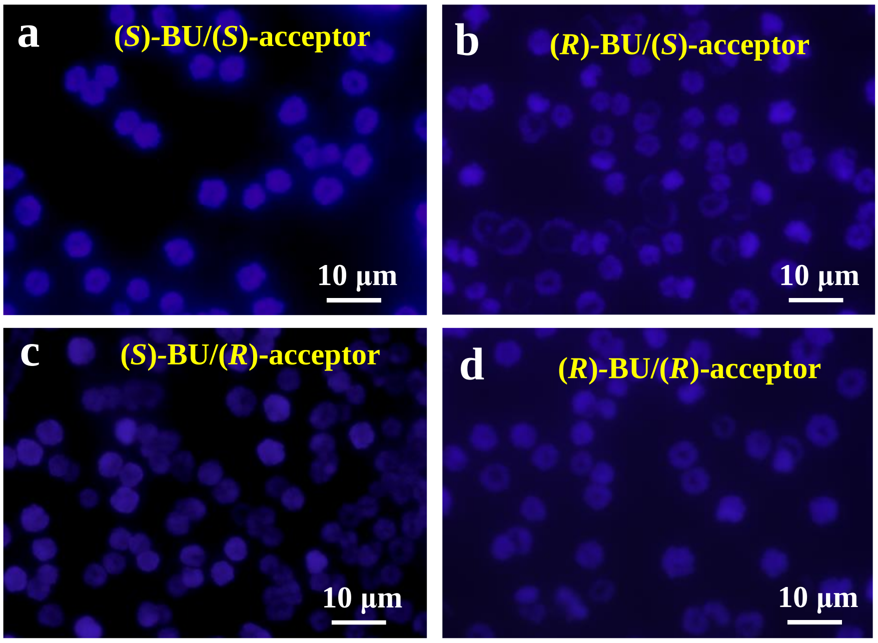

Figure S35. Fluorescent microscopy images of different combination co-assemblies. **a**, (*S*)-BU/(*S*)-acceptor. **b**, (*R*)-BU/(*S*)-acceptor. **c**, (*S*)-BU/(*R*)-acceptor. **d**, (*R*)-BU/(*R*)-acceptor. Acceptor molar ratio is 40 %. [BU] = 4 mM in methanol.

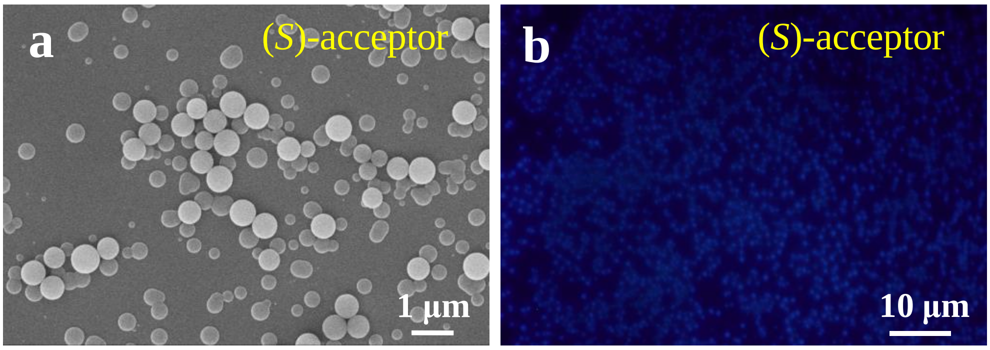

Figure S36. **a**, SEM and **b**, fluorescent microscopy images of (*S*)-acceptor interfacial assemblies. [(*S*)-acceptor] = 1.6 mM in MeOH.

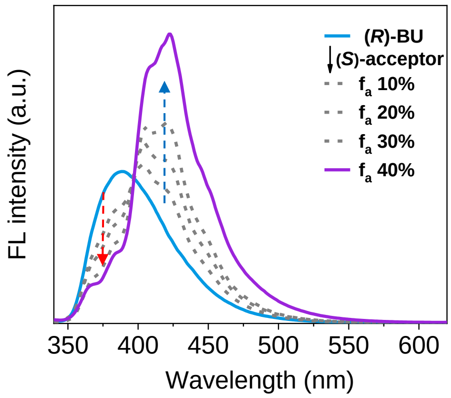

Figure S37. Energy transfer revealed by fluorescence spectra of (*R*)-BU/(*S*)-acceptor co-assemblies with increased acceptor fractions. [BU] = 4 mM in methanol.

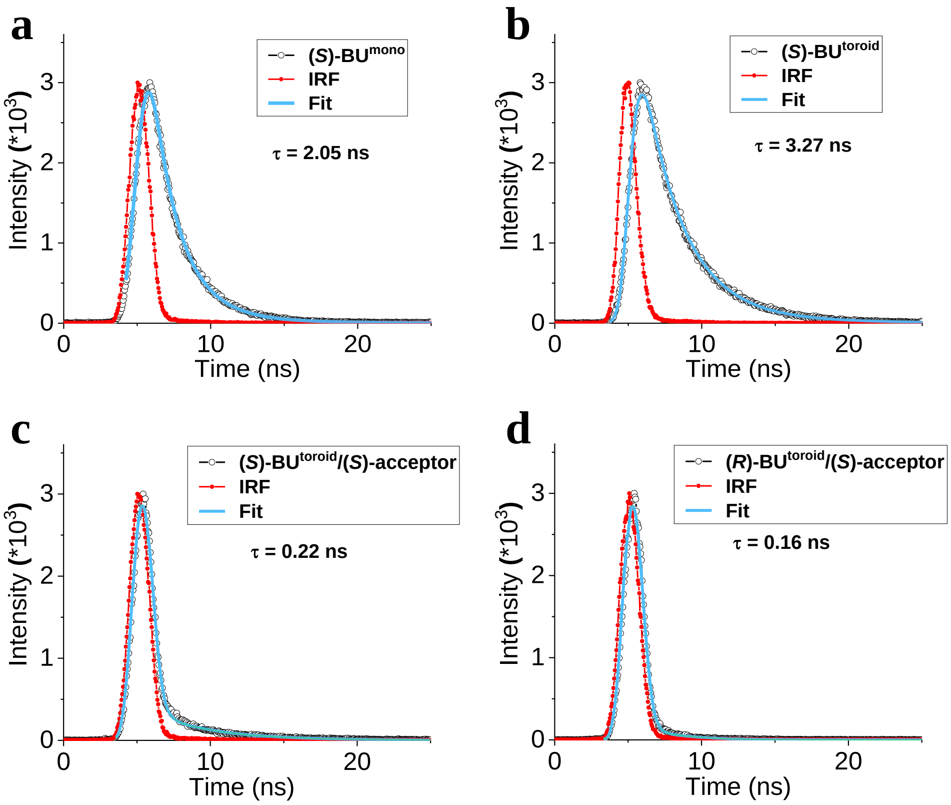

Figure S38. Emission decay curves of **a**, (*S*)-BU monomer in dilute MeOH. **b**, (*S*)-BU micro-toroids. **c**, (*S*)-BU/(*S*)-acceptor co-assemblies. **d**, (*R*)-BU/(*S*)-acceptor co-assemblies. Emission wavelength decay at 390 nm. τ represents average fluorescence lifetime. The energy transfer efficiency was calculated according to the equation of Φ_{ET} = 1-τ_{DA}/τ_{D}, where τ_{DA} and τ_{D} were the lifetime of donor BU in the presence and absence of acceptor dye, respectively.

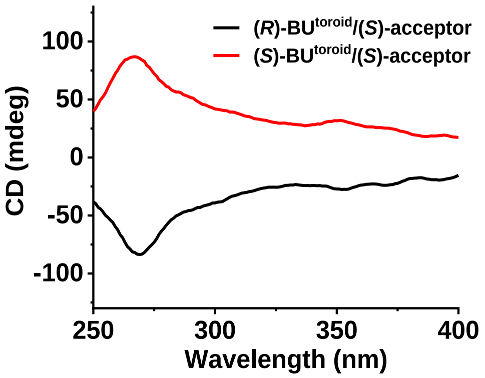

Figure S39. CD spectrum of (*R*)-BU/(*S*)-acceptor and (*S*)-BU/(*S*)-acceptor interfacial co-assemblies. Acceptor molar ratio is 40 %. [BU] = 4 mM in methanol.

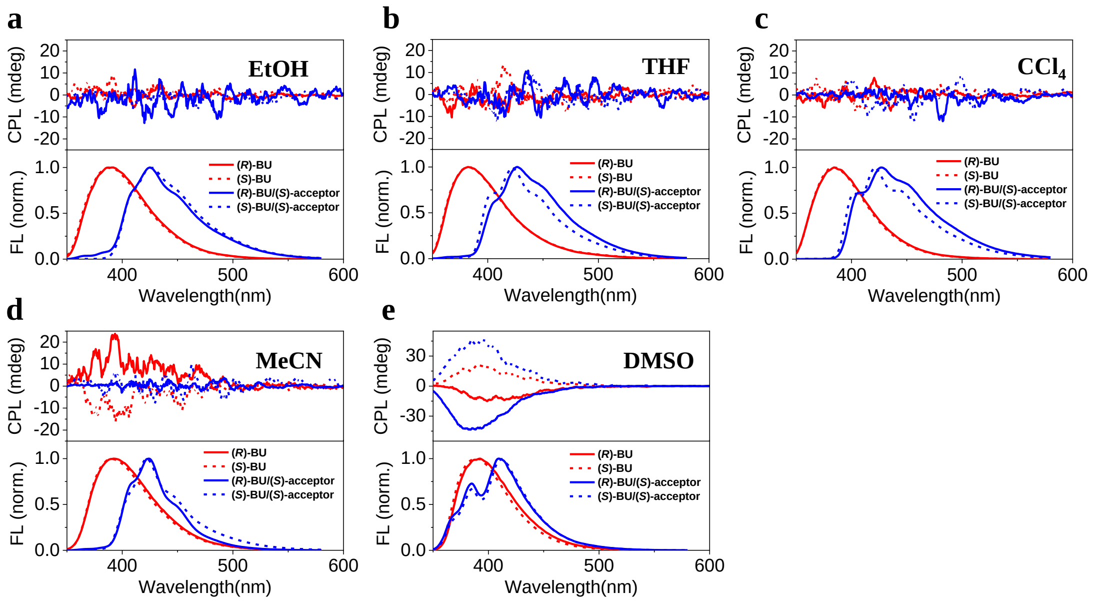

Figure S40. Emission and CPL spectra of BU interfacial self-assemblies (red lines) and BU/acceptor interfacial co-assemblies (blue lines) by using different solvents. **a**, EtOH. **b**, THF. **c**, CCl_4. **d**, MeCN. **e**, DMSO. [BU] = 4 mM in all the solvents, BU/acceptor = 5:2. λ_{ex} = 300 nm.

Efficient energy transfer occurred in all these interfacial co-assemblies except of DMSO. However, in EtOH, THF and CCl_4, both BU assemblies and BU/acceptor co-assemblies did not show any CPL signals (Fig. S40a-c, upper lines). In MeCN and DMSO, the BU interfacial assemblies exhibited CPL activity (red lines, Fig. S40d-e), but energy transfer-mediated CPL emission ascribed to the acceptor dye was suppressed. The CPL emission (centered near 390 nm) of BU/acceptor interfacial co-assemblies in DMSO system was ascribed to the donor BU (Fig. S40e, upper blue lines).

6. **Additional spectra.**

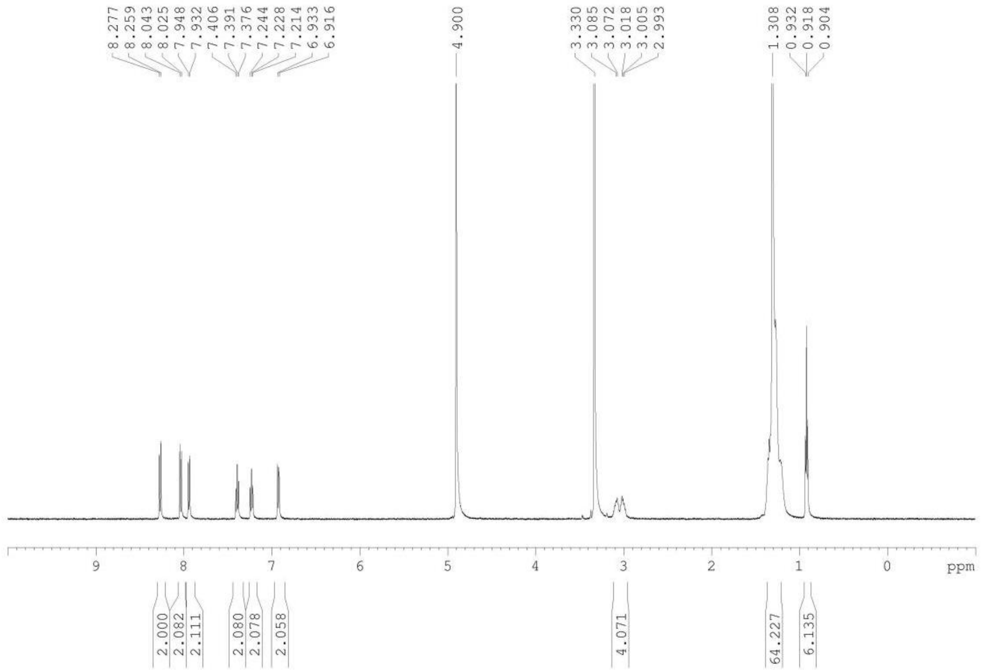

Figure S41. ^{1}H-NMR spectrum of (*S*)-BU (500 MHz, CD_3OD, 293 K).

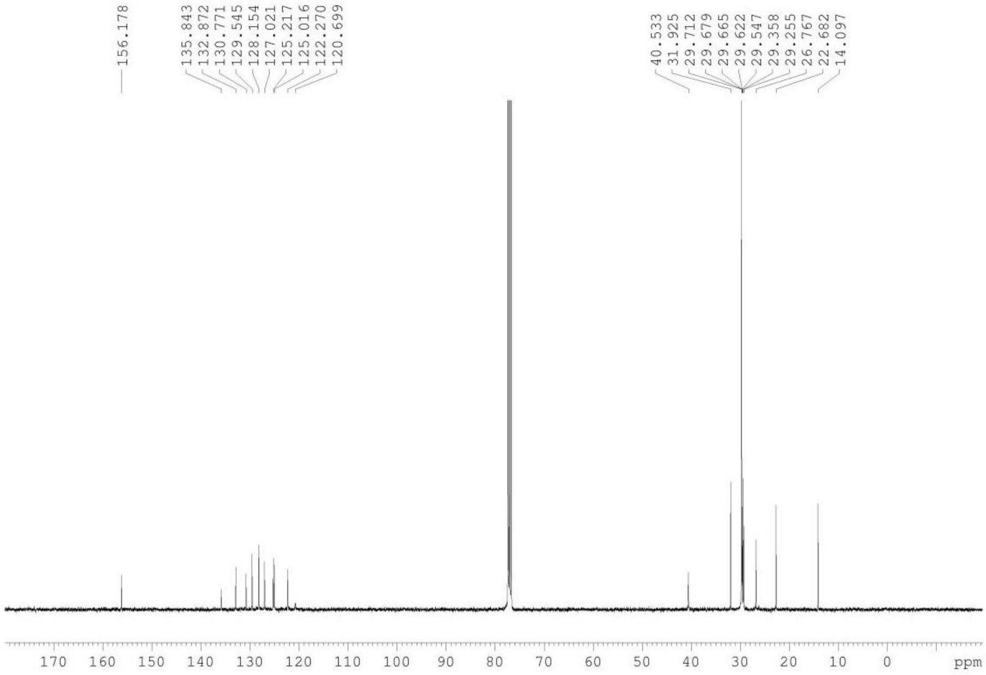

Figure S42. ^{13}C-NMR spectrum of (*S*)-BU (100 MHz, $CDCl_3$, 293 K).

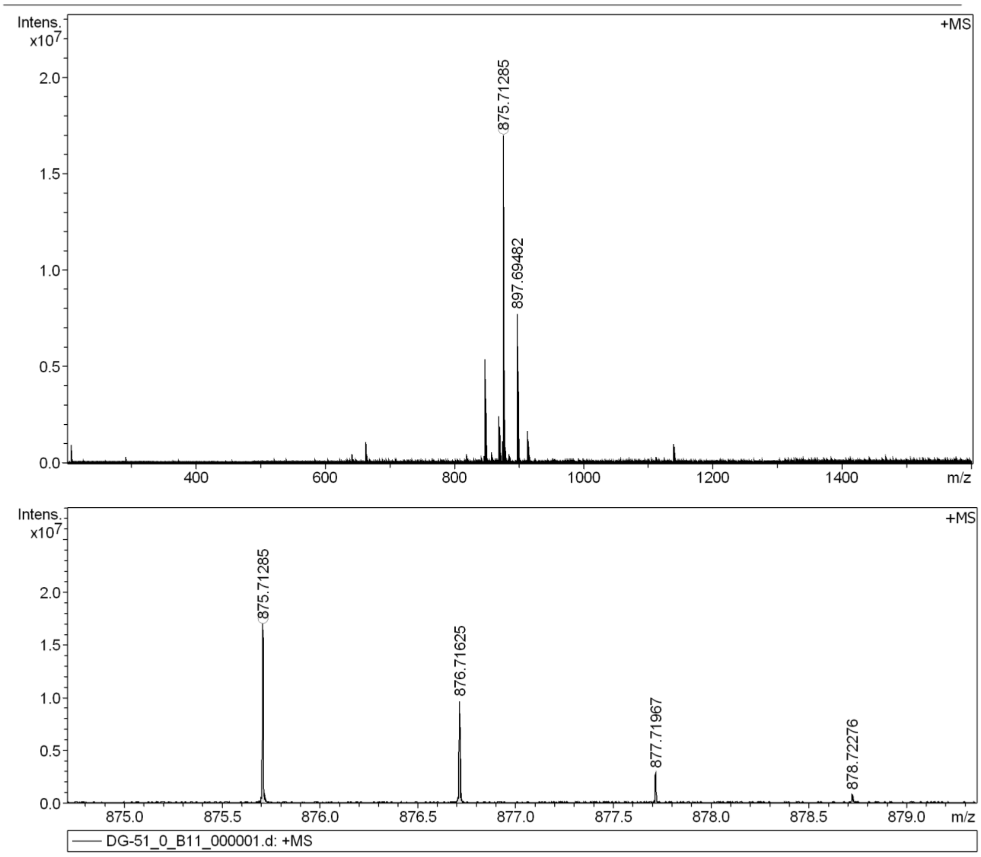

Meas. m/z	#	Ion Formula	Score	m/z	err [ppm]	Mean err [ppm]	mSigma	rdb	e⁻ Conf	N-Rule
875.712850	1	C58H91N4O2	100.00	875.713655	-0.9	0.8	42.7	15.5	even	ok

Figure S43. The MALDI-TOF MS spectrum of (*S*)-BU.

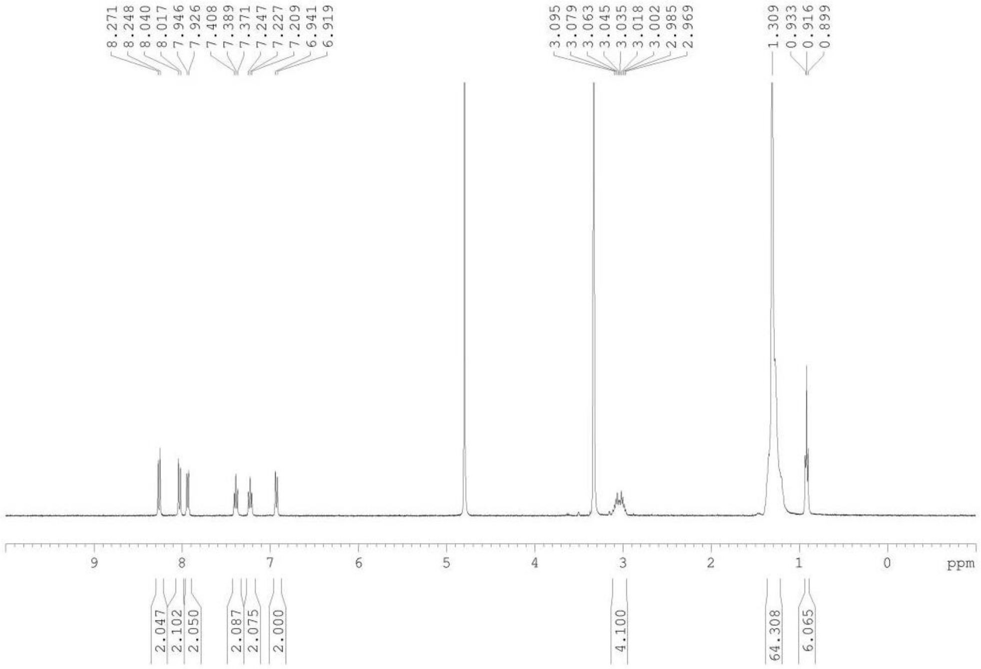

Figure S44. ^{1}H-NMR spectrum of (*R*)-BU (400 MHz, CD_3OD, 293 K).

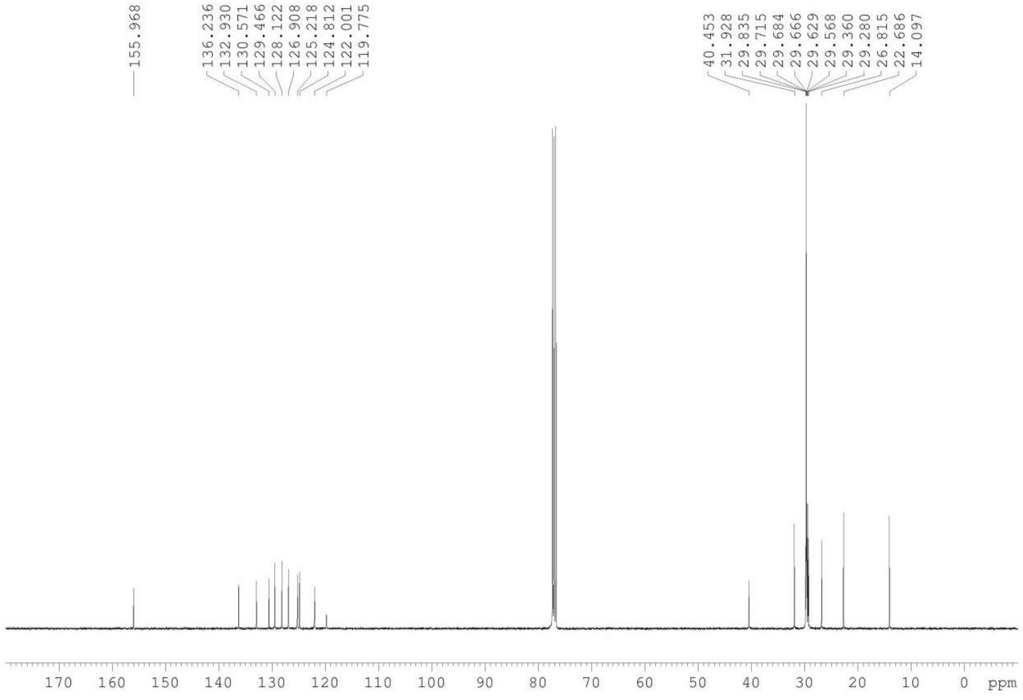

Figure S45. ^{13}C-NMR spectrum of (*R*)-BU (100 MHz, $CDCl_3$, 293 K).

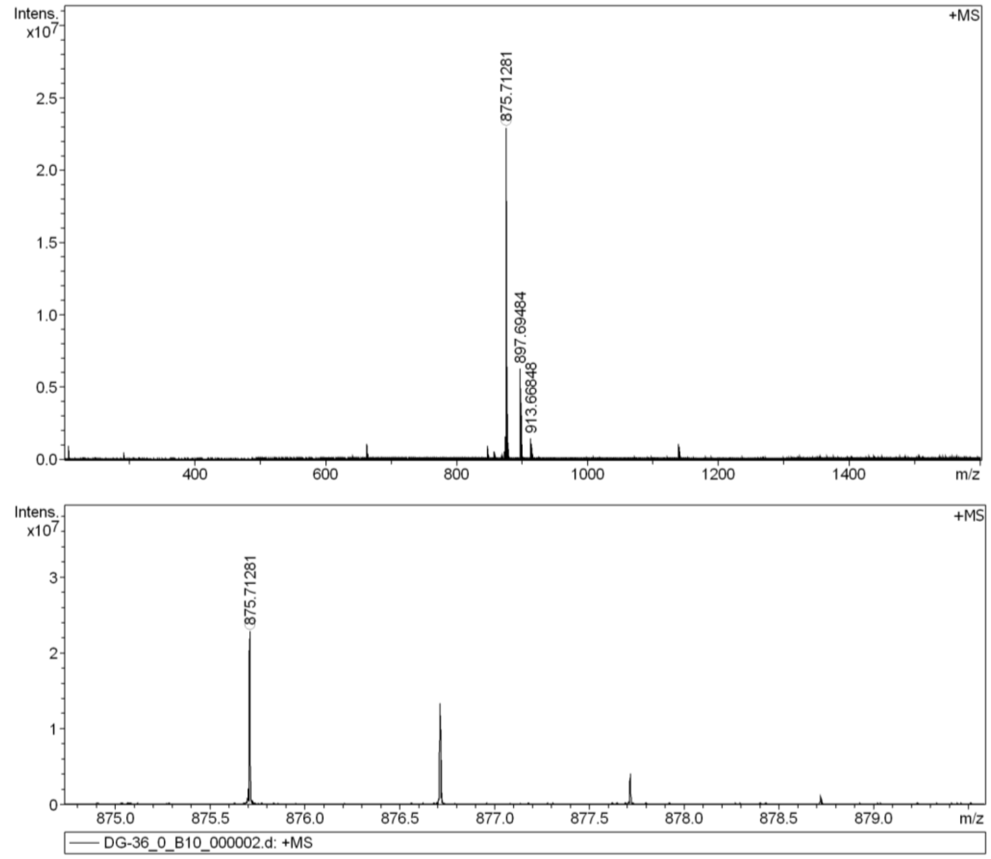

Meas. m/z	#	Ion Formula	Score	m/z	err [ppm]	Mean err [ppm]	mSigma	rdb	e⁻ Conf	N-Rule
875.712811	1	$C_{58}H_{91}N_4O_2$	100.00	875.713655	-1.0	0.9	35.0	15.5	even	ok

Figure S46. The MALDI-TOF MS spectrum of (*R*)-BU.

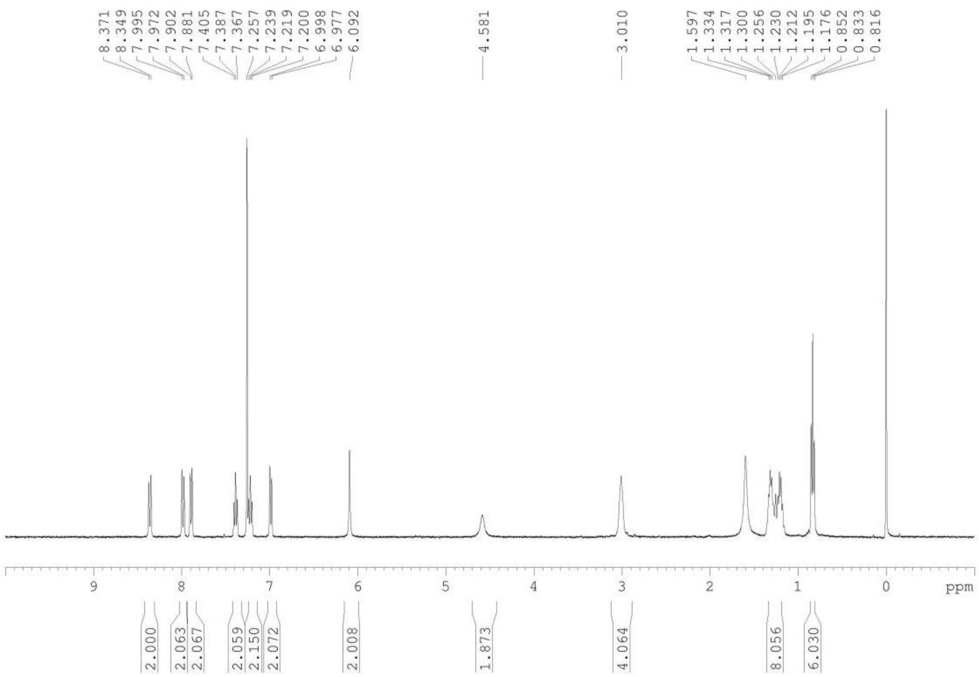

Figure S47. ^{1}H-NMR spectrum of *rac*-BU (300 MHz, $CDCl_3$, 293 K).

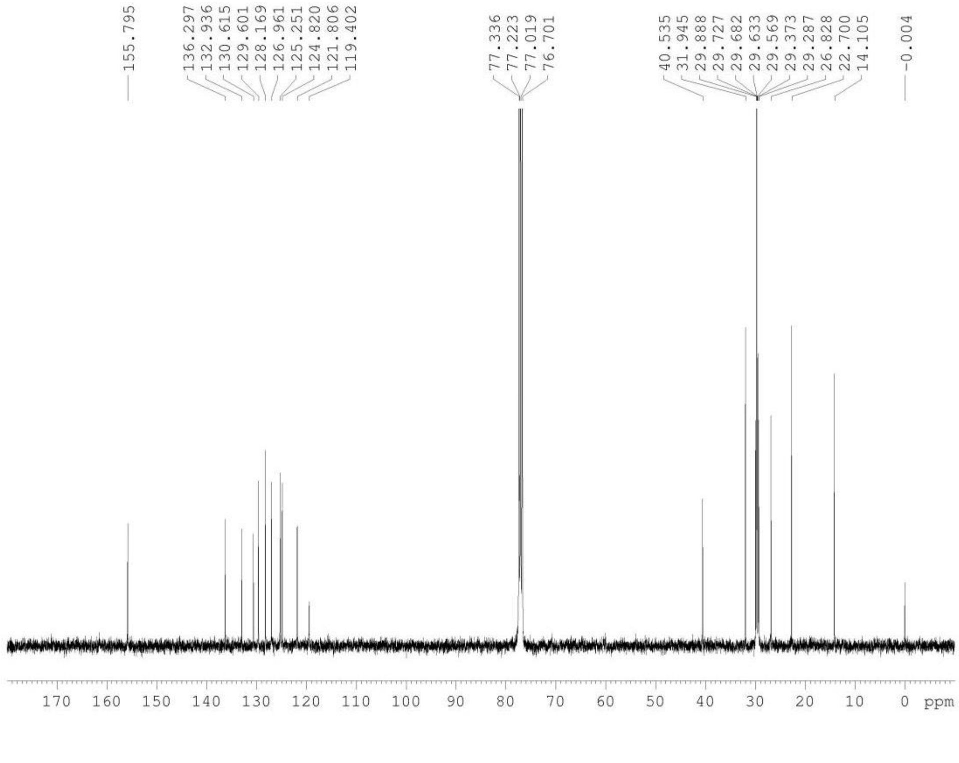

Figure S48. ^{13}C-NMR spectrum of *rac*-BU (100 MHz, $CDCl_3$, 293 K).

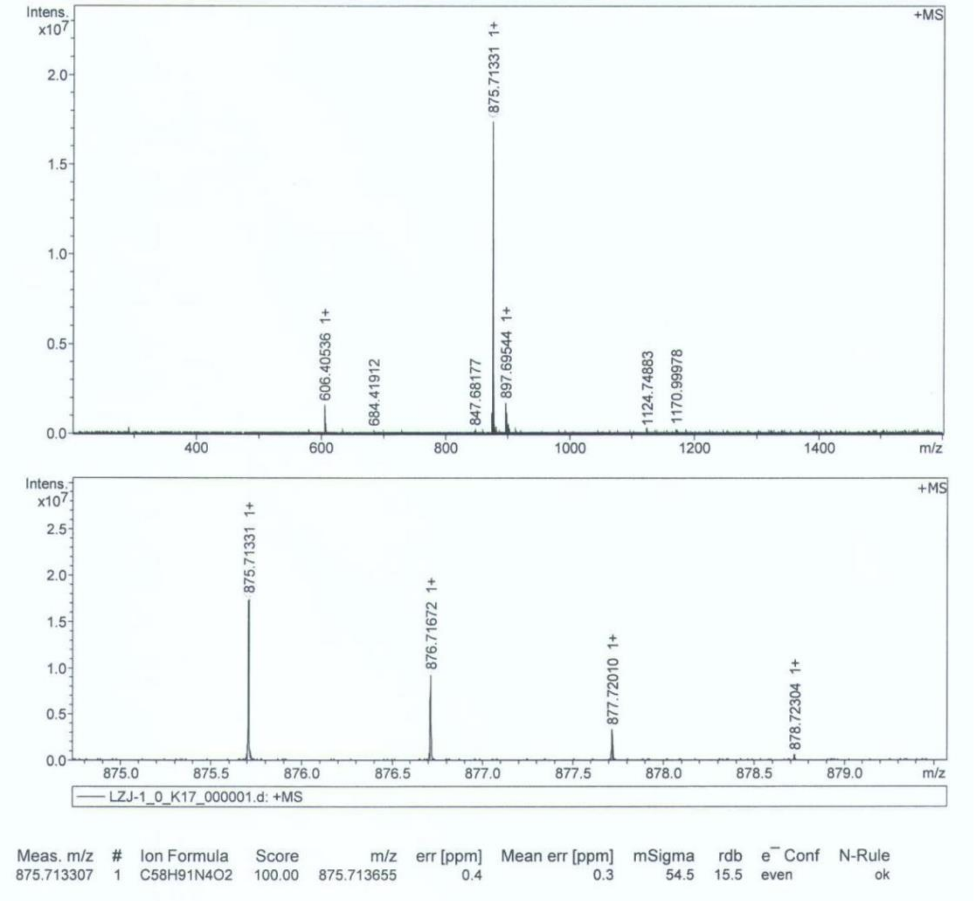

Figure S49. The MALDI-TOF MS spectrum of *rac*-BU.

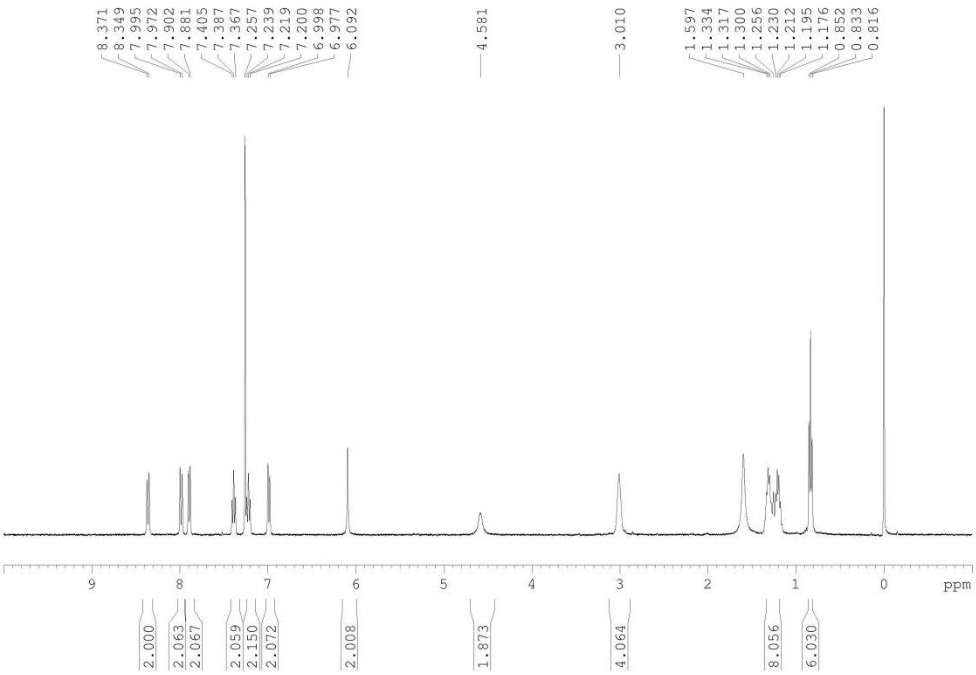

Figure S50. ^{1}H-NMR spectrum of (*S*)-BU-C4 (400 MHz, $CDCl_3$, 293 K).

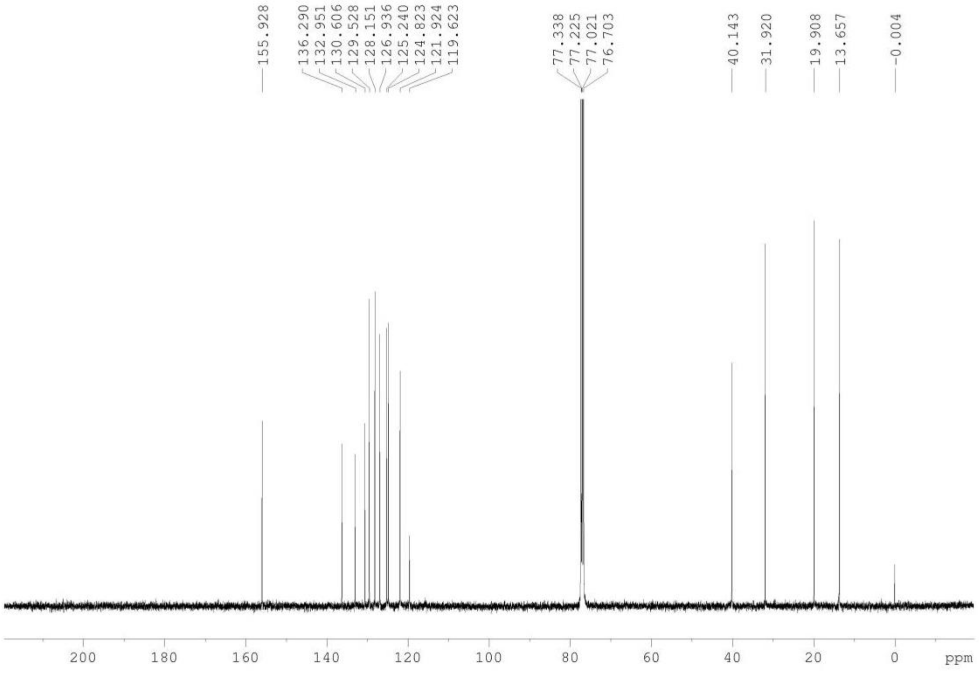

Figure S51. ^{13}C-NMR spectrum of (*S*)-BU-C4 (100 MHz, $CDCl_3$, 293 K).

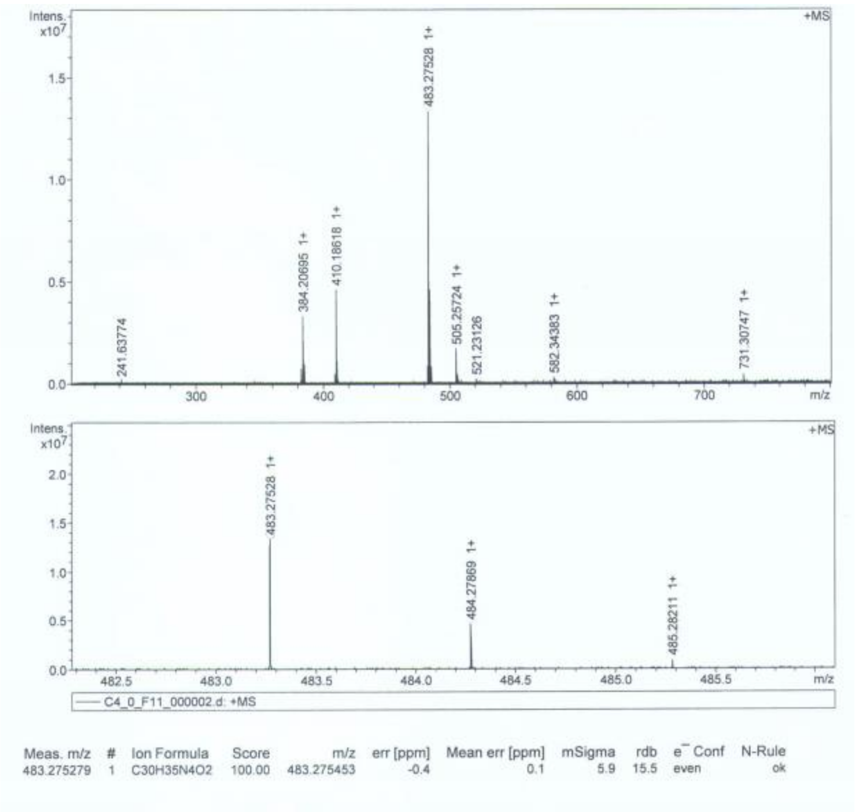

Figure S52. The MALDI-TOF MS spectrum of (*S*)-BU-C4.

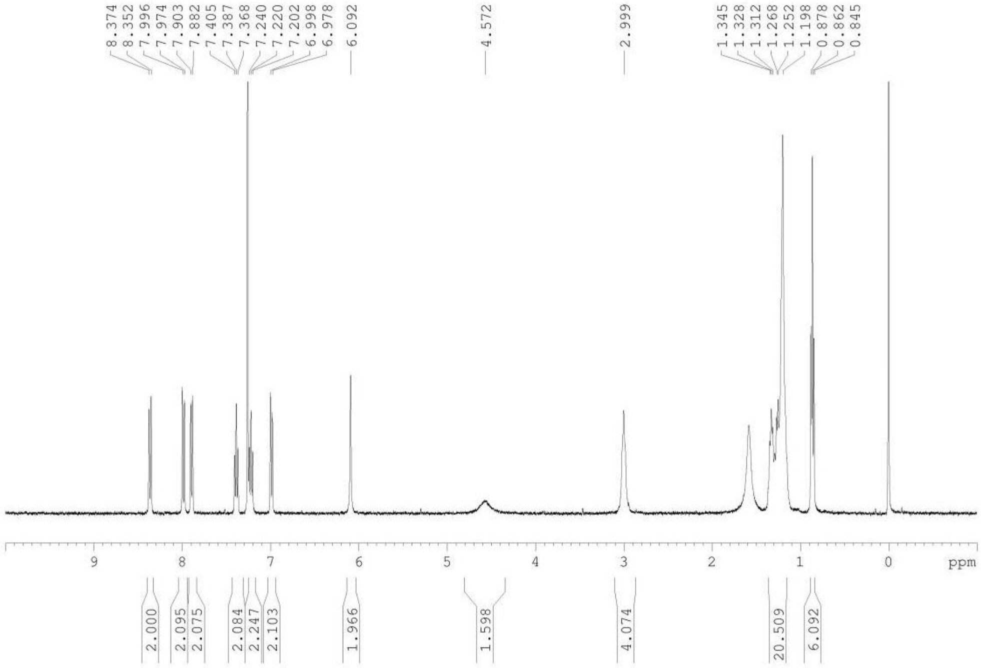

Figure S53. ^{1}H-NMR spectrum of (*S*)-BU-C7 (400 MHz, $CDCl_3$, 293 K).

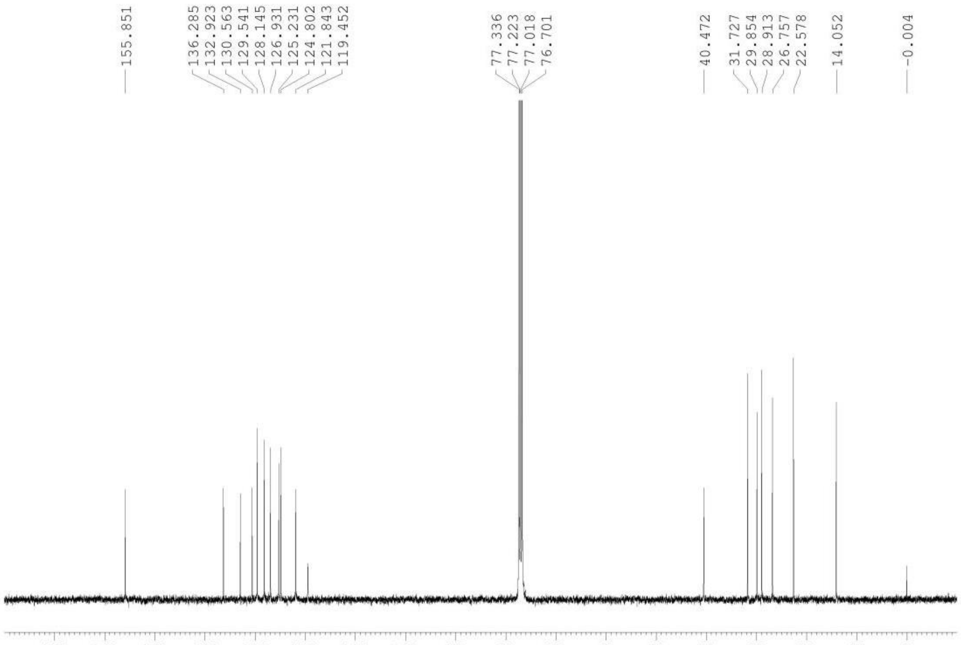

Figure S54. ^{13}C-NMR spectrum of (*S*)-BU-C7 (100 MHz, $CDCl_3$, 293 K).

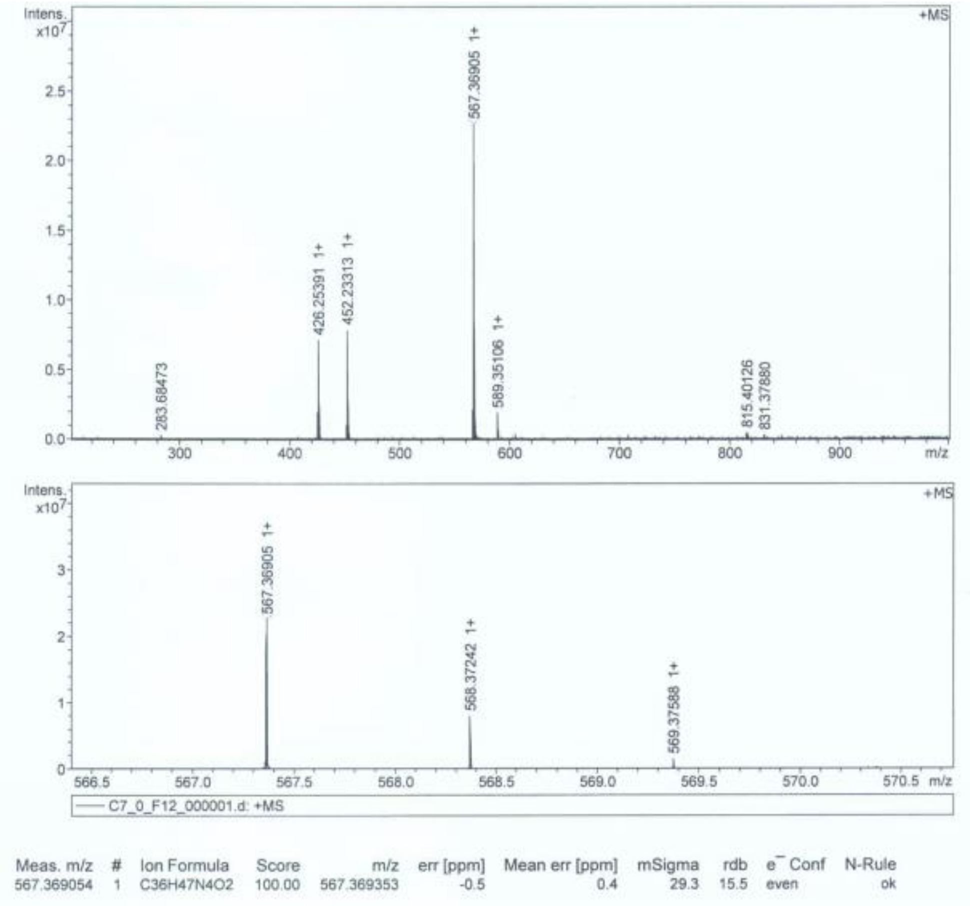

Figure S55. The MALDI-TOF MS spectrum of (*S*)-BU-C7.

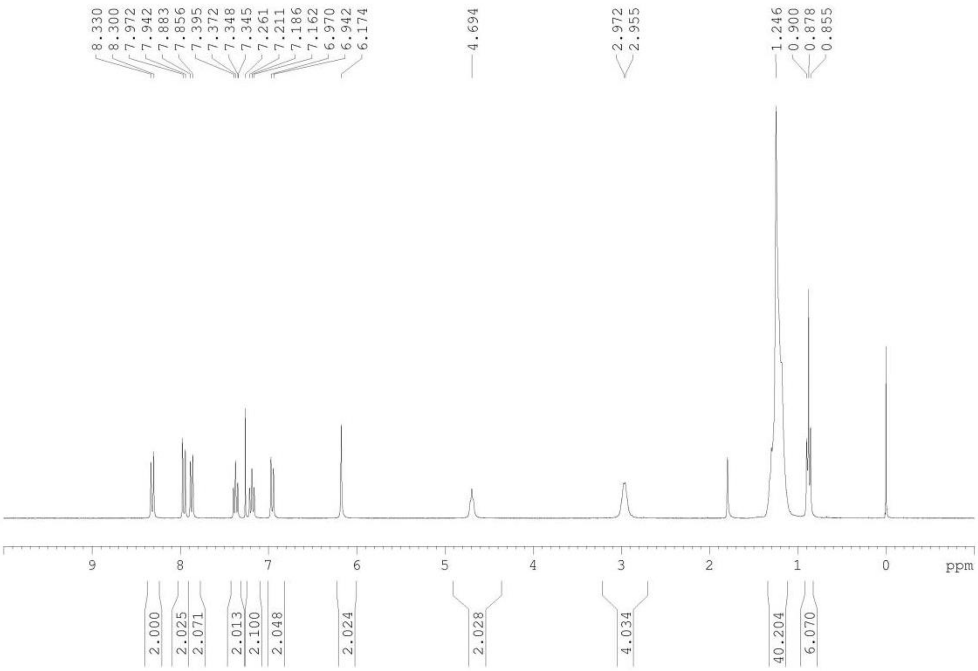

Figure S56. ^{1}H-NMR spectrum of (*S*)-BU-C12 (300 MHz, $CDCl_3$, 293 K).

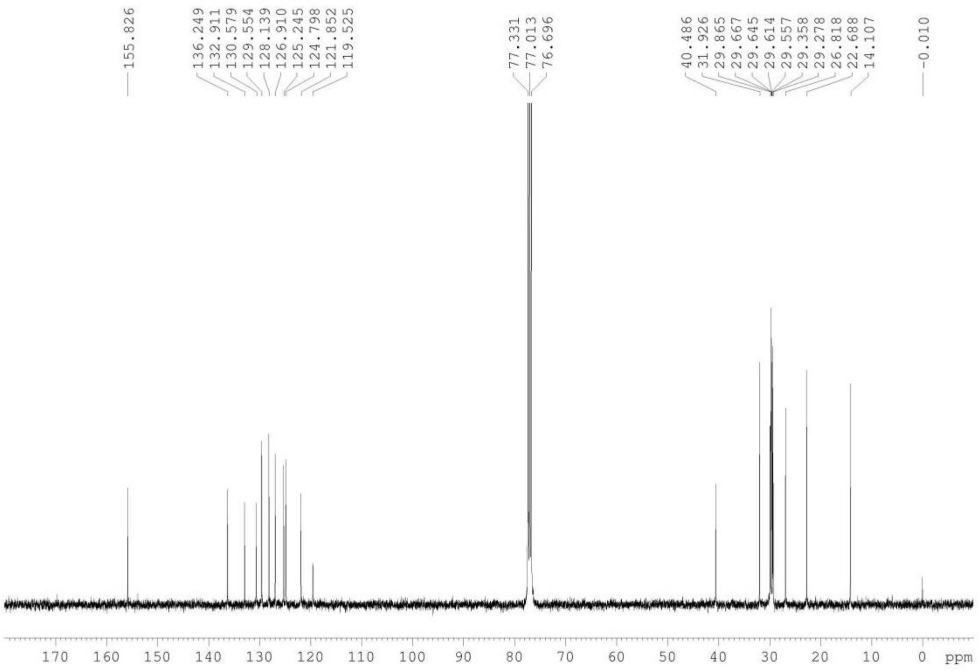

Figure S57. ^{13}C-NMR spectrum of (*S*)-BU-C12 (100 MHz, $CDCl_3$, 293 K).

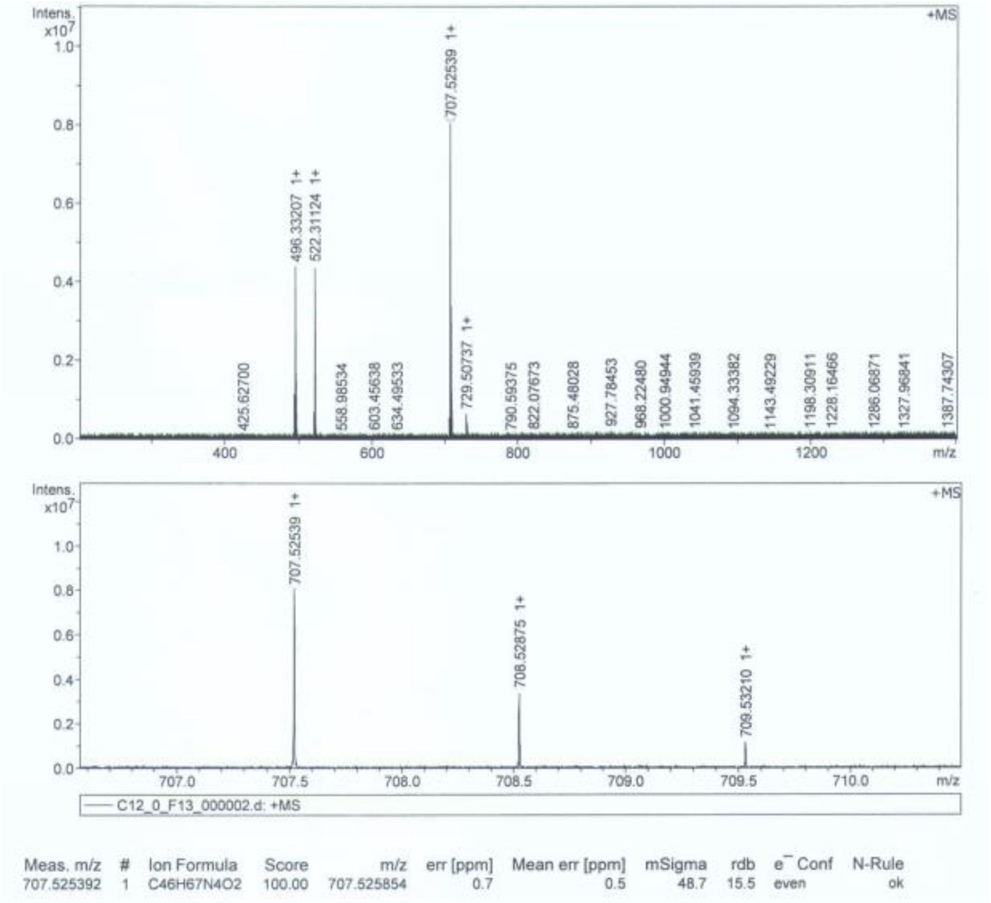

Figure S58. The MALDI-TOF MS spectrum of (*S*)-BU-C12.

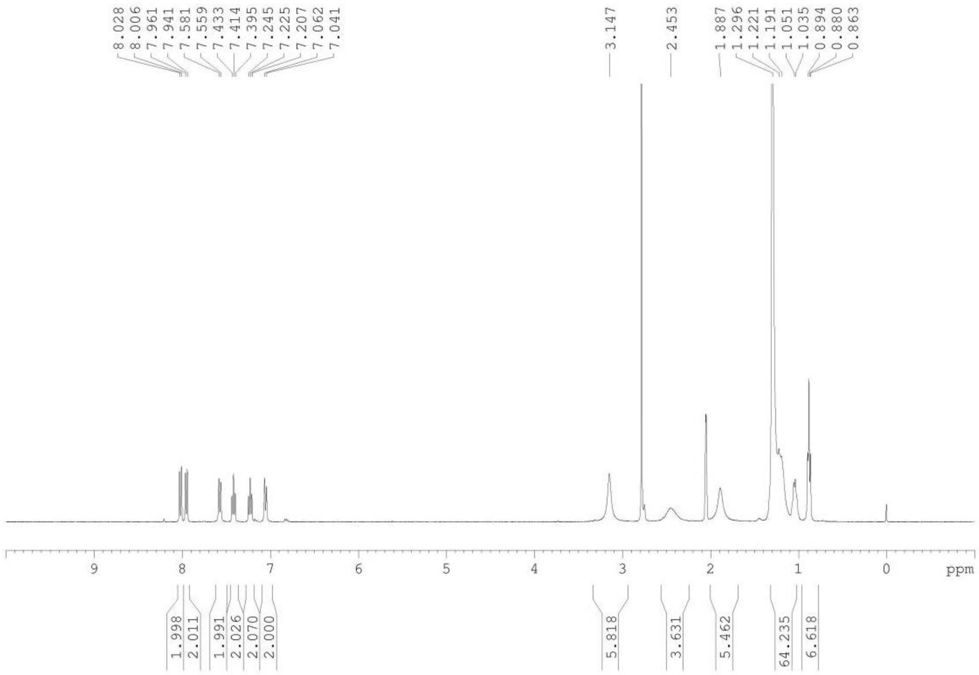

Figure S59. ^{13}C-NMR spectrum of (*S*)-BU-Me (400 MHz, Acetone-D_6, 293 K).

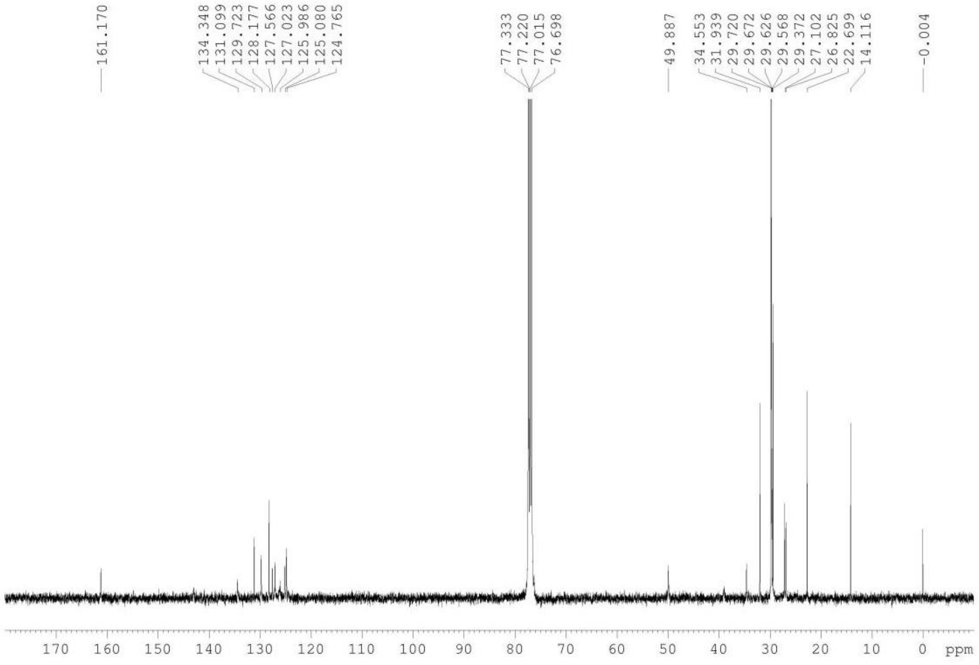

Figure S60. ^{13}C-NMR spectrum of (*S*)-BU-Me (100 MHz, $CDCl_3$, 293 K).

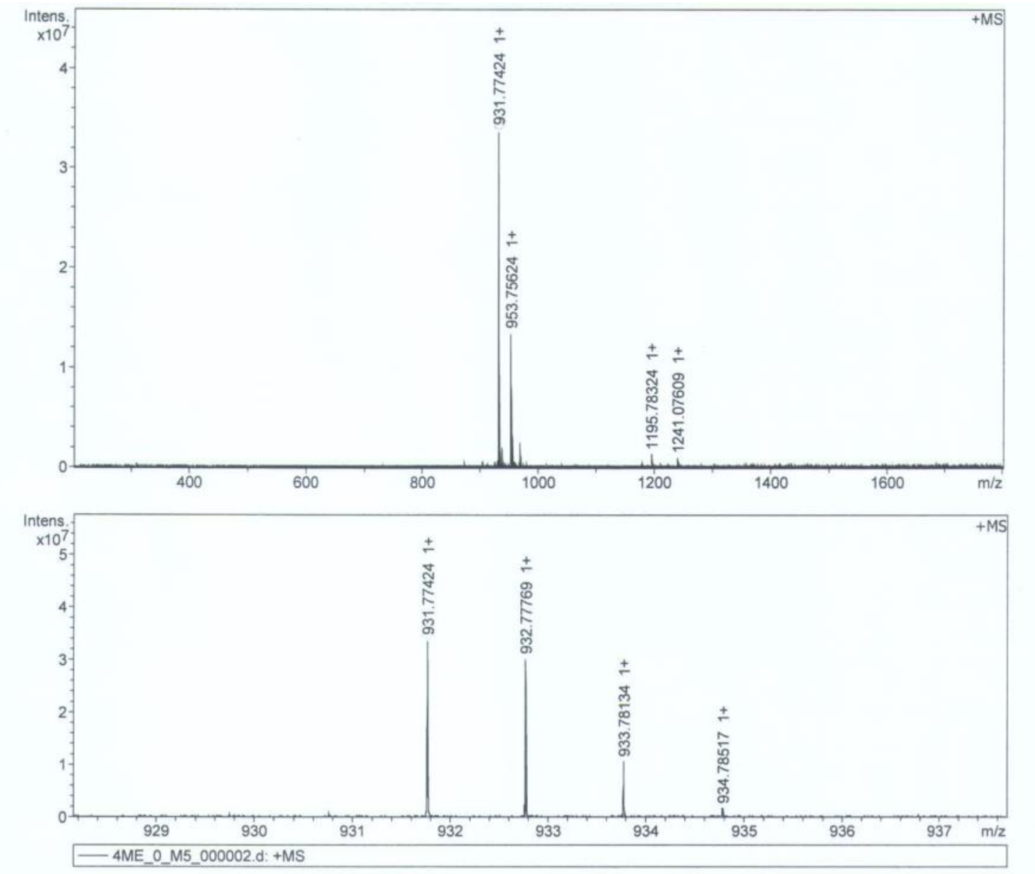

Meas. m/z	#	Ion Formula	Score	m/z	err [ppm]	Mean err [ppm]	mSigma	rdb	e⁻ Conf	N-Rule
931.774245	1	C62H99N4O2	100.00	931.776255	2.2	2.0	92.6	15.5	even	ok

Figure S61. The MALDI-TOF MS spectrum of (*S*)-BU-Me.

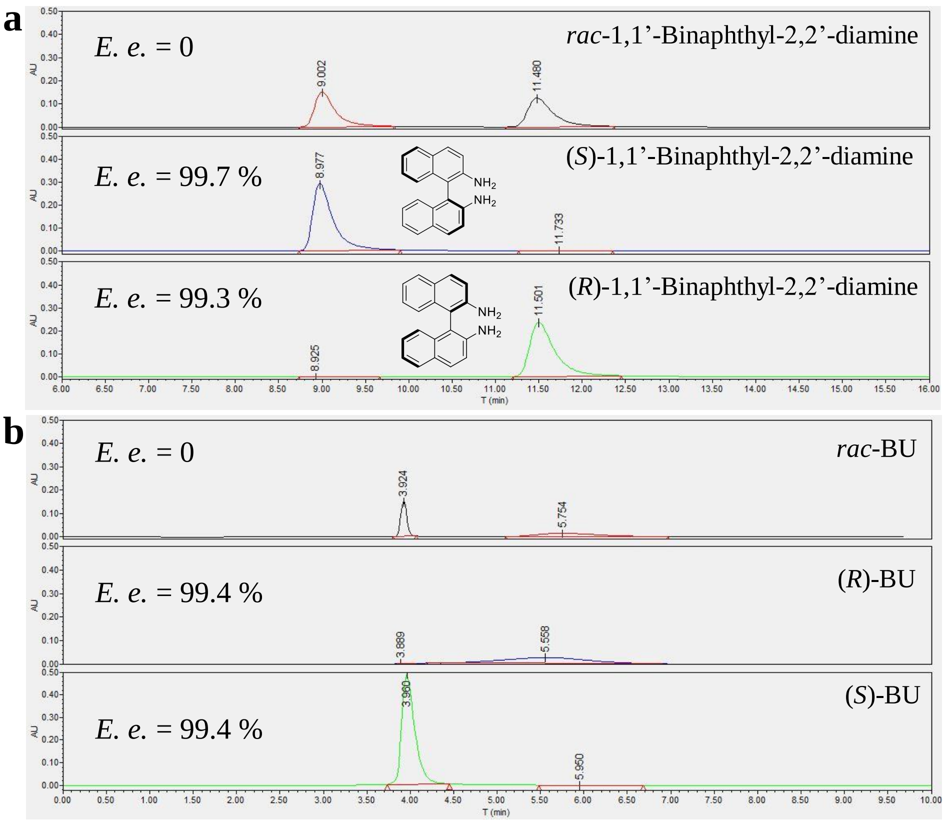

Figure S62. HPLC chromatograms of **a**, Starting material binaphthyl-diamine enantiomers and their racemates. **b**, BU enantiomers and their racemates. Measured on Waters chromatographic apparatus by an analytical chiral column CHIRALPAK AD-H, 5um, eluent is isopropanol/hexane, v/v=1:25, flow rate is 0.78 mL/min, 293 K.

7. Single crystal data

Table S1. Crystal data and structure refinement for (*S*)-BU and *rac*-BU.

	(*S*)-BU	*rac*-BU
Identification code	CCDC 2203258	CCDC 2203259
Empirical formula	C60H96N4O3S	C58H90N4O2
Formula weight	953.46	875.33
Temperature/K	169.99(13)	170.00(16)
Crystal system	triclinic	monoclinic
Space group	P1	P21/c
a/Å	8.82910(10)	24.8537(3)
b/Å	8.83350(10)	9.55700(10)
c/Å	38.9149(3)	23.3953(3)
α/°	85.0230(10)	90
β/°	86.1590(10)	108.7850(10)
γ/°	68.7860(10)	90
Volume/Å3	2816.64(5)	5261.01(11)
Z	2	4
ρcalcg/cm3	1.124	1.105
μ/mm1	0.854	0.5
F(000)	1048	1928
Crystal size/mm3	0.1 × 0.1 × 0.01	0.3 × 0.03 × 0.02
Radiation	Cu Kα (λ = 1.54184)	Cu Kα (λ = 1.54184)
2Θ range for data collection/°	4.562 to 151.278	7.514 to 151.334
Index ranges	-10 ≤ h ≤ 11, -11 ≤ k ≤ 11, -48 ≤ l ≤ 47	-31 ≤ h ≤ 30, -10 ≤ k ≤ 11, -29 ≤ l ≤ 28
Reflections collected	85336	116178
Independent reflections	21114 [Rint= 0.0598, Rsigma= 0.0486]	10621 [Rint= 0.0529, Rsigma= 0.0248]
Data/restraints/parameters	21114/3/1233	10621/0/579
Goodness-of-fit on F2	1.033	1.034
Final R indexes [I>=2σ (I)]	R1= 0.0561, wR2= 0.1519	R1= 0.0442, wR2= 0.1154
Final R indexes [all data]	R1= 0.0725, wR2= 0.1651	R1= 0.0579, wR2= 0.1260
Largest diff. peak/hole / e Å-3	0.42/-0.45	0.25/-0.30
