# Supplementary material for: Hierarchically self-assembled homochiral helical microtoroids
Source: Nat Nanotechnol. 2022 Nov 3;17(12):1294–302. doi: 10.1038/s41565-022-01234-w (PMC9747612; doi:10.1038/s41565-022-01234-w)
Supplement: Supplementary file 3 — CheckCIF file of the (S)-BU crystal. [file 41565_2022_1234_MOESM3_ESM.pdf]

## checkCIF (basic structural check) running

*Checking for embedded fcf data in CIF ...*
*Found embedded fcf data in CIF. Extracting fcf data from uploaded CIF, please wait* . . . . . . . . . . . .

## checkCIF/PLATON (basic structural check)

Structure factors have been supplied for datablock(s) tx5332

THIS REPORT IS FOR GUIDANCE ONLY. IF USED AS PART OF A REVIEW PROCEDURE FOR PUBLICATION, IT SHOULD NOT REPLACE THE EXPERTISE OF AN EXPERIENCED CRYSTALLOGRAPHIC REFEREE.

No syntax errors found. CIF dictionary
Please wait while processing .... Interpreting this report

Structure factor report

# Datablock: tx5332

Bond precision: C-C = 0.0060 A Wavelength=1.54184

Cell: a=8.8291(1) b=8.8335(1) c=38.9149(3)
alpha=85.023(1) beta=86.159(1) gamma=68.786(1)

Temperature: 170 K

| | Calculated | Reported |
|---|---|---|
| Volume | 2816.64(5) | 2816.64(5) |
| Space group | P 1 | P 1 |
| Hall group | P 1 | P 1 |
| Moiety formula | C58 H90 N4 O2, C2 H6 O S | C2 H6 O S, C58 H90 N4 O2 |
| Sum formula | C60 H96 N4 O3 S | C60 H96 N4 O3 S |
| Mr | 953.47 | 953.46 |
| Dx,g cm-3 | 1.124 | 1.124 |
| Z | 2 | 2 |
| Mu (mm-1) | 0.854 | 0.854 |
| F000 | 1048.0 | 1048.0 |
| F000' | 1051.23 | |
| h,k,lmax | 11,11,48 | 11,11,48 |
| Nref | 23392[ 11696] | 21114 |
| Tmin,Tmax | 0.918,0.991 | 0.712,1.000 |
| Tmin' | 0.918 | |

Correction method= # Reported T Limits: Tmin=0.712 Tmax=1.000 AbsCorr = MULTI-SCAN

Data completeness= 1.81/0.90 Theta(max)= 75.639

R(reflections)= 0.0561( 16603) wR2(reflections)= 0.1651( 21114)

S = 1.033 Npar= 1233

The following ALERTS were generated. Each ALERT has the format
**test-name_ALERT_alert-type_alert-level**.
Click on the hyperlinks for more details of the test.

## Alert level C

PLAT042_ALERT_1_C Calc. and Reported MoietyFormula Strings Differ Please Check
PLAT244_ALERT_4_C Low 'Solvent' Ueq as Compared to Neighbors of S Check
PLAT244_ALERT_4_C Low 'Solvent' Ueq as Compared to Neighbors of S1 Check
PLAT260_ALERT_2_C Large Average Ueq of Residue Including S1 0.102 Check
PLAT340_ALERT_3_C Low Bond Precision on C-C Bonds ............... 0.00596 Ang.
PLAT415_ALERT_2_C Short Inter D-H..H-X H1AA ..H5LB . 2.08 Ang.
x,1+y,z = 1_565 Check
PLAT415_ALERT_2_C Short Inter D-H..H-X H5AA ..H5B . 2.06 Ang.
1+x,y,z = 1_655 Check
PLAT911_ALERT_3_C Missing FCF Refl Between Thmin & STh/L= 0.600 4 Report
PLAT987_ALERT_1_C The Flack x is >> 0 - Do a BASF/TWIN Refinement Please Check

## Alert level G

PLAT007_ALERT_5_G Number of Unrefined Donor-H Atoms ............. 8 Report
PLAT033_ALERT_4_G Flack x Value Deviates > 3.0 * sigma from Zero . 0.035 Note
PLAT072_ALERT_2_G SHELXL First Parameter in WGHT Unusually Large 0.11 Report
PLAT154_ALERT_1_G The s.u.'s on the Cell Angles are Equal ..(Note) 0.001 Degree
PLAT720_ALERT_4_G Number of Unusual/Non-Standard Labels .......... 293 Note
PLAT767_ALERT_4_G INS Embedded LIST 6 Instruction Should be LIST 4 Please Check
PLAT910_ALERT_3_G Missing # of FCF Reflection(s) Below Theta(Min). 1 Note
PLAT912_ALERT_4_G Missing # of FCF Reflections Above STh/L= 0.600 546 Note
PLAT933_ALERT_2_G Number of HKL-OMIT Records in Embedded .res File 2 Note
PLAT978_ALERT_2_G Number C-C Bonds with Positive Residual Density. 2 Info
PLAT992_ALERT_5_G Repd & Actual _reflns_number_gt Values Differ by 3 Check

0 **ALERT level A** = Most likely a serious problem - resolve or explain
0 **ALERT level B** = A potentially serious problem, consider carefully
9 **ALERT level C** = Check. Ensure it is not caused by an omission or oversight
11 **ALERT level G** = General information/check it is not something unexpected

3 ALERT type 1 CIF construction/syntax error, inconsistent or missing data
6 ALERT type 2 Indicator that the structure model may be wrong or deficient
3 ALERT type 3 Indicator that the structure quality may be low
6 ALERT type 4 Improvement, methodology, query or suggestion
2 ALERT type 5 Informative message, check

It is advisable to attempt to resolve as many as possible of the alerts in all categories. Often the minor alerts point to easily fixed oversights, errors and omissions in your CIF or refinement strategy, so attention to these fine details can be worthwhile. In order to resolve some of the more serious problems it may be necessary to carry out additional measurements or structure refinements. However, the purpose of your study may justify the reported deviations and the more serious of these should normally be commented upon in the discussion or experimental section of a paper or in the "special_details" fields of the CIF. checkCIF was carefully designed to identify outliers and unusual parameters, but every test has its limitations and alerts that are not important in a particular case may appear. Conversely, the absence of alerts does not guarantee there are no aspects of the results needing attention. It is up to the individual to critically assess their own results and, if necessary, seek expert advice.

**Publication of your CIF in IUCr journals**

A basic structural check has been run on your CIF. These basic checks will be run on all CIFs submitted for publication in IUCr journals (*Acta Crystallographica*, *Journal of Applied Crystallography*, *Journal of Synchrotron Radiation*); however, if you intend to submit to *Acta Crystallographica Section C* or *E* or *IUCrData*, you should make sure that full publication checks are run on the final version of your CIF prior to submission.

**Publication of your CIF in other journals**

Please refer to the *Notes for Authors* of the relevant journal for any special instructions relating to CIF submission.

**PLATON version of 18/05/2022; check.def file version of 17/05/2022**

# **Datablock tx5332** - ellipsoid plot

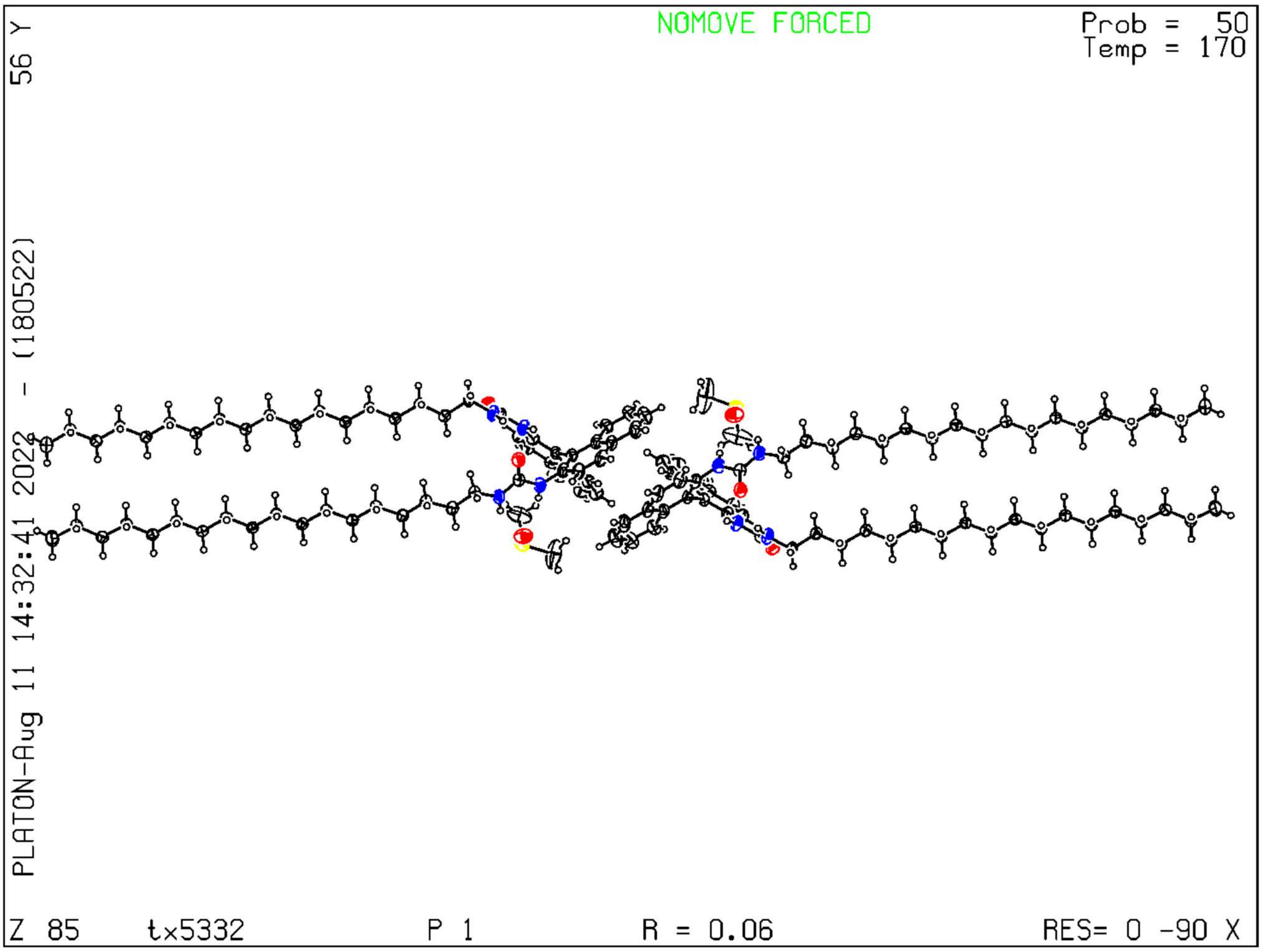


---

Download CIF editor (publCIF) from the IUCr
Download CIF editor (enCIFer) from the CCDC
Test a new CIF entry
