# Supplementary material for: Hierarchically self-assembled homochiral helical microtoroids
Source: Nat Nanotechnol. 2022 Nov 3;17(12):1294–302. doi: 10.1038/s41565-022-01234-w (PMC9747612; doi:10.1038/s41565-022-01234-w)
Supplement: Supplementary file 4 — CheckCIF file of the rac-BU crystal. [file 41565_2022_1234_MOESM4_ESM.pdf]

checkCIF (basic structural check) running

Checking for embedded fcf data in CIF ...
Found embedded fcf data in CIF. Extracting fcf data from uploaded CIF, please wait . . . . . .

checkCIF/PLATON (basic structural check)

Structure factors have been supplied for datablock(s) tx2674

THIS REPORT IS FOR GUIDANCE ONLY. IF USED AS PART OF A REVIEW PROCEDURE FOR PUBLICATION, IT SHOULD NOT REPLACE THE EXPERTISE OF AN EXPERIENCED CRYSTALLOGRAPHIC REFEREE.

No syntax errors found. CIF dictionary
Please wait while processing .... Interpreting this report

Structure factor report

Datablock: tx2674

Bond precision:	C-C = 0.0021 A		Wavelength=1.54184
Cell:	a=24.8537(3)	b=9.5570(1)	c=23.3953(3)
	alpha=90	beta=108.785(1)	gamma=90
Temperature: 170 K			

	Calculated	Reported
Volume	5261.01(11)	5261.01(11)
Space group	P 21/c	P 1 21/c 1
Hall group	-P 2ybc	-P 2ybc
Moiety formula	C58 H90 N4 O2	C58 H90 N4 O2
Sum formula	C58 H90 N4 O2	C58 H90 N4 O2
Mr	875.34	875.33
Dx,g cm-3	1.105	1.105
Z	4	4
Mu (mm-1)	0.500	0.500
F000	1928.0	1928.0
F000'	1932.80	
h,k,lmax	31,12,29	31,11,29
Nref	10923	10621
Tmin,Tmax	0.982,0.990	0.728,1.000
Tmin'	0.861	

Correction method= # Reported T Limits: Tmin=0.728 Tmax=1.000 AbsCorr = MULTI-SCAN

Data completeness= 0.972 Theta(max)= 75.667

R(reflections)= 0.0442(8499) wR2(reflections)= 0.1260(10621)

S = 1.034 Npar= 579

The following ALERTS were generated. Each ALERT has the format
test-name_ALERT_alert-type_alert-level.
Click on the hyperlinks for more details of the test.

Alert level C

PLAT420_ALERT_2_C D-H Bond Without Acceptor N3 --H3 . Please Check
PLAT911_ALERT_3_C Missing FCF Refl Between Thmin & STh/L= 0.600 15 Report

Alert level G

PLAT007_ALERT_5_G Number of Unrefined Donor-H Atoms .... 4 Report
PLAT767_ALERT_4_G INS Embedded LIST 6 Instruction Should be LIST 4 Please Check
PLAT910_ALERT_3_G Missing # of FCF Reflection(s) Below Theta(Min). 1 Note
PLAT912_ALERT_4_G Missing # of FCF Reflections Above STh/L= 0.600 274 Note
PLAT955_ALERT_1_G Reported (CIF) and Actual (FCF) Lmax Differ by . 1 Units
PLAT978_ALERT_2_G Number C-C Bonds with Positive Residual Density. 9 Info
PLAT992_ALERT_5_G Repd & Actual _reflns_number_gt Values Differ by 2 Check

0 **ALERT level A** = Most likely a serious problem - resolve or explain
0 **ALERT level B** = A potentially serious problem, consider carefully
2 **ALERT level C** = Check. Ensure it is not caused by an omission or oversight
7 **ALERT level G** = General information/check it is not something unexpected

1 ALERT type 1 CIF construction/syntax error, inconsistent or missing data
2 ALERT type 2 Indicator that the structure model may be wrong or deficient
2 ALERT type 3 Indicator that the structure quality may be low
2 ALERT type 4 Improvement, methodology, query or suggestion
2 ALERT type 5 Informative message, check

It is advisable to attempt to resolve as many as possible of the alerts in all categories. Often the minor alerts point to easily fixed oversights, errors and omissions in your CIF or refinement strategy, so attention to these fine details can be worthwhile. In order to resolve some of the more serious problems it may be necessary to carry out additional measurements or structure refinements. However, the purpose of your study may justify the reported deviations and the more serious of these should normally be commented upon in the discussion or experimental section of a paper or in the "special_details" fields of the CIF. checkCIF was carefully designed to identify outliers and unusual parameters, but every test has its limitations and alerts that are not important in a particular case may appear. Conversely, the absence of alerts does not guarantee there are no aspects of the results needing attention. It is up to the individual to critically assess their own results and, if necessary, seek expert advice.

Publication of your CIF in IUCr journals

A basic structural check has been run on your CIF. These basic checks will be run on all CIFs submitted for publication in IUCr journals (*Acta Crystallographica*, *Journal of Applied Crystallography*, *Journal of Synchrotron Radiation*); however, if you intend to submit to *Acta Crystallographica Section C* or *E* or *IUCrData*, you should make sure that full publication checks are run on the final version of your CIF prior to submission.

Publication of your CIF in other journals

Please refer to the *Notes for Authors* of the relevant journal for any special instructions relating to CIF submission.

PLATON version of 18/05/2022; check.def file version of 17/05/2022

Datablock tx2674 - ellipsoid plot

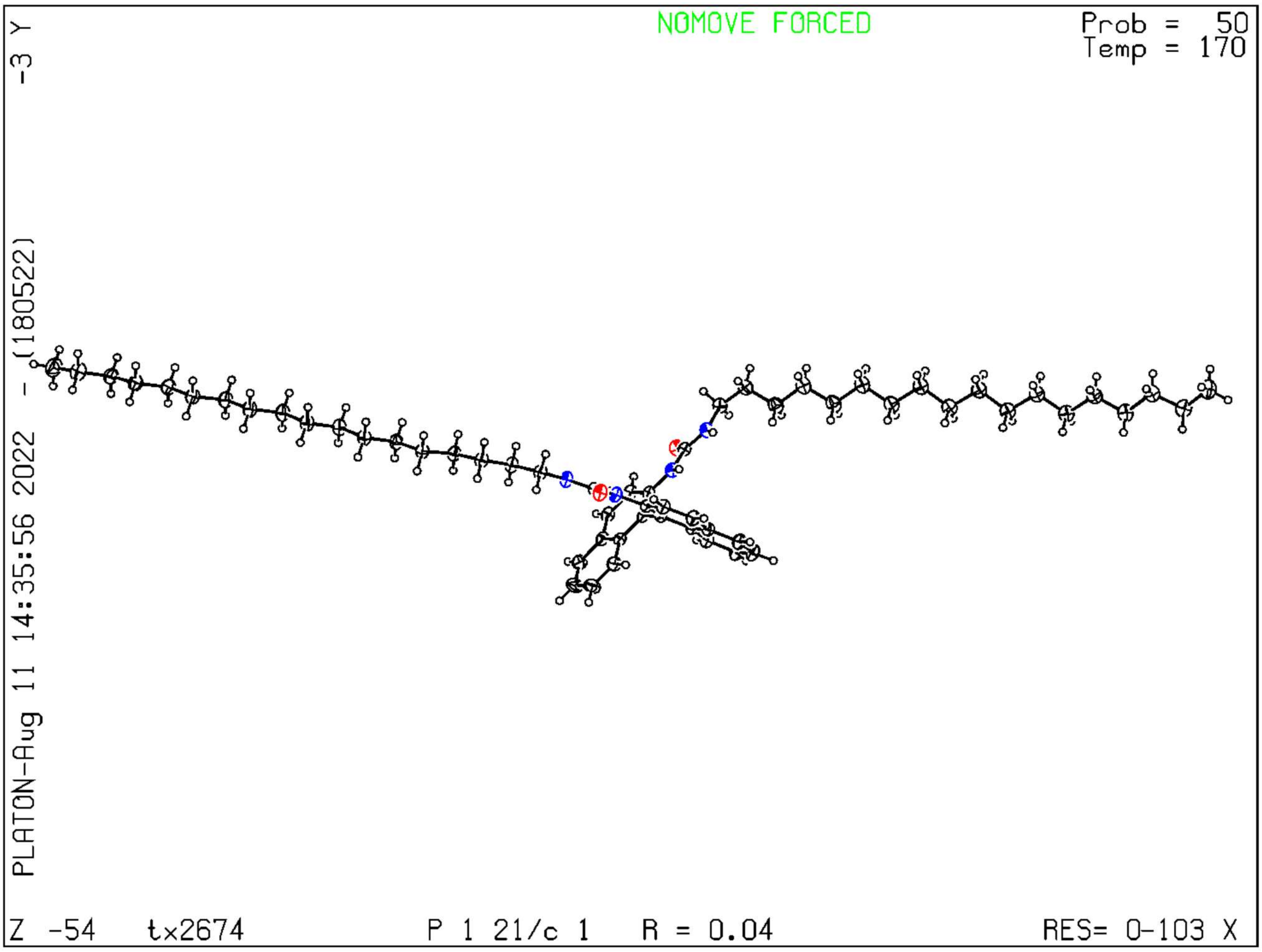

Download CIF editor (publCIF) from the IUCr
Download CIF editor (enCIFer) from the CCDC
Test a new CIF entry
